# The *Rhododendron* Genome and Chromosomal Organization Provide Insight into Shared Whole-Genome Duplications across the Heath Family (Ericaceae)

**DOI:** 10.1093/gbe/evz245

**Published:** 2019-11-18

**Authors:** Valerie L Soza, Dale Lindsley, Adam Waalkes, Elizabeth Ramage, Rupali P Patwardhan, Joshua N Burton, Andrew Adey, Akash Kumar, Ruolan Qiu, Jay Shendure, Benjamin Hall

**Affiliations:** 1 Department of Biology, University of Washington, Seattle, WA; 2 Department of Genome Sciences, University of Washington, Seattle, WA; 3 Brotman Baty Institute for Precision Medicine, Seattle, WA; 4 Howard Hughes Medical Institute, University of Washington, Seattle, WA; 5 Department of Laboratory Medicine, University of Washington, Seattle, WA; 6 Adaptive Biotechnologies, Seattle, WA; 7 Department of Molecular and Medical Genetics, Oregon Health and Science University, Portland, OR; 8 Department of Pediatrics, Stanford University, Palo Alto, CA; 9 Retired

**Keywords:** chromatin conformation capture (Hi-C), chromosome-scale scaffolding, de novo genome assembly, linkage map, restriction-site associated DNA (RAD) sequencing, synteny

## Abstract

The genus *Rhododendron* (Ericaceae), which includes horticulturally important plants such as azaleas, is a highly diverse and widely distributed genus of >1,000 species. Here, we report the chromosome-scale de novo assembly and genome annotation of *Rhododendron williamsianum* as a basis for continued study of this large genus. We created multiple short fragment genomic libraries, which were assembled using ALLPATHS-LG. This was followed by contiguity preserving transposase sequencing (CPT-seq) and fragScaff scaffolding of a large fragment library, which improved the assembly by decreasing the number of scaffolds and increasing scaffold length. Chromosome-scale scaffolding was performed by proximity-guided assembly (LACHESIS) using chromatin conformation capture (Hi-C) data. Chromosome-scale scaffolding was further refined and linkage groups defined by restriction-site associated DNA (RAD) sequencing of the parents and progeny of a genetic cross. The resulting linkage map confirmed the LACHESIS clustering and ordering of scaffolds onto chromosomes and rectified large-scale inversions. Assessments of the *R. williamsianum* genome assembly and gene annotation estimate them to be 89% and 79% complete, respectively. Predicted coding sequences from genome annotation were used in syntenic analyses and for generating age distributions of synonymous substitutions/site between paralgous gene pairs, which identified whole-genome duplications (WGDs) in *R. williamsianum*. We then analyzed other publicly available Ericaceae genomes for shared WGDs. Based on our spatial and temporal analyses of paralogous gene pairs, we find evidence for two shared, ancient WGDs in *Rhododendron* and *Vaccinium* (cranberry/blueberry) members that predate the Ericaceae family and, in one case, the Ericales order.

## Introduction

Scientific interest in the plant genus *Rhododendron* L., which includes azaleas, derives from the great morphological diversification that accompanied speciation and geographic dispersal of *Rhododendron* species across Eurasia, North America, and the Malesian archipelago (Irving and Hebda 1993). Within the northern temperate zone, *Rhododendron* species have adapted to grow in virtually every montane area (Irving and Hebda 1993). In Southeast Asia, similar dispersal and adaptation have accompanied major tectonic mountain-building events (Hall 1998). In addition, species within this genus are valued for their floral and vegetative diversity and are widely cultivated across Asia, North America, and Europe (Xing et al. 2017), with >35,000 cultivars produced around the world (Leslie 2004, 2017; McDonald S, personal communication).

The widely accepted classification scheme for rhododendrons (Sleumer 1980; Chamberlain 1996) names eight subgenera, of which four account for the vast majority of species: subg. *Hymenanthes* (Blume) K. Koch, *Pentanthera* (G. Don) Poyarkova, *Rhododendron*, and *Tsutsusi* (Sweet) Pojark. The plant species at the center of this project, *Rhododendron williamsianum* Rehder and E.H. Wilson, belongs to the subgenus *Hymenanthes*, a large taxon with representatives on all Northern Hemisphere continents (Chamberlain 1982). The rhododendrons of subgenus *Hymenanthes* are evergreen and lack leaf scales (elepidote). Since rhododendrons in India and China first came to the attention of European plant explorers (ca. AD 1800), elepidote species have been widely planted and hybridized with one another to produce new varieties (Irving and Hebda 1993).

Recent developments in genomics make it possible for many organisms to receive intensive genetic study comparable in depth to that achieved in familiar model organisms (e.g., *Arabidopsis*, *Caenorhabditis*, *Drosophila*). The experiments, data, and conclusions we present here are intended to bring *Rhododendron* to this forefront of genomics by sequencing and chromosome-scale scaffolding of the nuclear genome of *R. williamsianum*. Knowledge of the content and chromosome location of these sequences enables genomic study of the many species that comprise genus *Rhododendron*.

*Rhododendron* is a member of the heath or heather family (Ericaceae), which contains >4,400 species and is an economically important family that includes cranberries, blueberries, huckleberries, and wintergreen. An important question raised by the availability of *Rhododendron* chromosome-level sequences is: how strictly conserved is the gross organization of the genome sequence across the Ericaceae, and how comparable is this genome to those of the other 4,400+ species in the family? One other diploid genome, *Vaccinium macrocarpon* Aiton (cranberry) (Polashock et al. 2014; Schlautman et al. 2017), is sequenced within the Ericaceae with scaffolds anchored to chromosomes, and two other draft, diploid genomes are available for *Rhododendron* and *Vaccinium* (Bian et al. 2014; Gupta et al. 2015; Zhang, Xu, et al. 2017). Both *Rhododendron* and *Vaccinium* represent species-rich groups within Ericaceae that diverged from one another ∼77 Ma (Rose et al. 2018), and have undergone multiple speciation shifts (Schwery et al. 2015; Rose et al. 2018). Chromosome evolution between these two genera represents the majority of the age of the family, which has a crown age of ∼90.5 Ma (Rose et al. 2018).

The central goal of our project has been to sequence the nuclear genome of *R. williamsianum* and order its sequences along chromosomes. To that end, we created DNA libraries for de novo assembly of DNA sequences using next generation methodologies. Building upon this resource, we employed LACHESIS assembly (Burton et al. 2013) with *R. williamsianum* Hi-C data (Lieberman-Aiden et al. 2009) to produce highly detailed physical maps of the *R. willliamsianum* chromosomes. We then carried out a genetic cross and analyzed the parents and progeny of this cross for the linkage relationships between genes on the 13 chromosomes. Alignment and reconciliation of the physical and genetic maps placed contiguous blocks of the assembled sequence precisely along the *R. williamsianum* chromosomes. With this chromosome-level genomic resource, we then examined genome evolution within the Ericaceae and uncovered multiple shared, ancient whole-genome duplications (WGDs).

## Materials and Methods

### *Rhododendron* Sampling

Rhododendrons of subgenus *Hymenanthes* were chosen as the focus of this research based upon their importance in horticulture, ready availability, and strict diploidy (2*n *=* *2*x *=* *26) (Janaki Ammal et al. 1950; Jones et al. 2007). Within subgenus *Hymenanthes*, the species *R. williamsianum* was chosen for genomic study for three reasons. First, we believe that its small area of wild occurrence might limit within-species genetic variation. Second, *R. williamsianum* has had widespread successful use as a parent in hybrid crosses, making it a species of interest among plant breeders. Third, we have local availability of a cultivated accession of this species bearing thousands of flower buds annually. *Rhododendron williamsianum* material used throughout this study was collected from the Rhododendron Species Botanical Garden, Federal Way, WA, USA (accession# 1966-606).

### Genomic Libraries

DNA for genomic libraries was extracted from *R. williamsianum* nuclei. Prior to DNA extraction, the nuclei were isolated from *R. williamsianum* floral buds using the method of Zhang et al. (1995) with minor variations (supplementary method SM1, Supplementary Material online).

Five *R. williamsianum* genomic libraries were constructed as recommended for use with ALLPATHS-LG (Gnerre et al. 2011; Ribeiro et al. 2012). These included one 180-bp short fragment library with overlapping reads, three 2-kb short jumping libraries, and one 29-kb insert fosmid library (supplementary table S1, Supplementary Material online). In addition, a high molecular weight genomic library was created for subsequent contiguity preserving transposase sequencing (CPT-seq) analysis (Amini et al. 2014). All libraries were sequenced on a HiSeq 2000 (Illumina, Inc.) using the standard paired-end sequencing protocol (Meyer and Kircher 2010).

### De Novo Assembly of Genomic Sequence

Next-generation sequencing data resulting from the five *R. williamsianum* libraries above (supplementary table S1, Supplementary Material online) were assembled using ALLPATHS-LG v40083 (Gnerre et al. 2011; Ribeiro et al. 2012) (supplementary method SM1, Supplementary Material online).

Data from CPT-seq were assembled by fragScaff v140324.1 (Adey et al. 2014) in an effort to improve the ALLPATHS-LG assembly prior to chromosome-scale scaffolding using contact probability maps and genetic linkage. We first created a masked assembly by mapping the initial ALLPATHS-LG assembly to itself using BLAST (Altschul et al. 1990), and removing regions of high similarity (≥85%). In this masked assembly, we also removed regions that aligned to known repeat elements in Repbase (Jurka et al. 2005), as well as regions with a level of shotgun coverage >3 SDs above the mean. fragScaff options were tailored to favor the scaffolding of smaller contigs that would typically be missed by the subsequent contact probability map scaffolding process (supplementary method SM1, Supplementary Material online). fragScaff was then run using the masked assembly and CPT-seq data.

### Proximity-Guided Chromosome-Scale Assembly

A proximity-based, short read library of *R. williamsianum* was constructed using the Hi-C protocol of Lieberman-Aiden et al. (2009) with minor modifications (supplementary method SM2, Supplementary Material online). The library was sequenced on a HiSeq 2000. Hi-C reads were aligned to the fragScaff scaffolds and combined using LACHESIS (Burton et al. 2013) (supplementary method SM2, Supplementary Material online).

### Linkage Map Analysis

In order to establish a linkage map of the *R. williamsianum* genome, we generated progeny from a genetic cross between the hybrid *R.* “Moonstone” and *R. campylocarpum*. Pollen of *R. campylocarpum* was applied to the stigmas of the hybrid R. “Moonstone” (*R. williamsianum*×*R. campylocarpum*). Seed capsules from this cross were harvested 5 months after pollination and stored at 4 °C in glassine envelopes. After storage in dry and cool conditions, seeds were released from their capsules and germinated on a layer of moss at 21 °C and high humidity for 12-h days under 600 W sodium vapor light. Seedlings were hardened off by gradually increasing the growth time at ambient temperature and humidity. One year after the cross was carried out, seedlings were planted in small pots. At ∼2 years of seedling age, DNA was extracted from progeny leaves using the DNeasy Plant Mini Kit (Qiagen) in a modified protocol (supplementary method SM3, Supplementary Material online).

For the linkage analysis, we created reduced complexity restriction-site associated DNA (RAD) sequencing libraries for the parents, *R. campylocarpum* and *R*. “Moonstone,” and for each of the 110 progeny. Each library was individually barcoded. The method of Etter et al. (2011) was employed for creating the RAD libraries with some modification (supplementary method SM4, Supplementary Material online) using the *PstI* restriction enzyme. Pooled libraries were paired-end sequenced on a HiSeq 2000.

From the RAD sequencing data, we removed low quality reads, reads with ambiguous barcodes or restriction sites, and their orphaned paired-end reads using process_radtags.pl (supplementary method SM4, Supplementary Material online) from Stacks v0.997 (Catchen et al. 2011, 2013). Data from the paired-end sequences allowed removal of PCR duplicates using samtools v0.1.18 (Li 2011) rmdup. Stacks denovo_map.pl (supplementary method SM4, Supplementary Material online) called the genotypes for parents and progeny and loaded the results into a MySQL database.

We chose RAD markers that showed SNP heterozygosity in parental genotypes and that were successfully genotyped by Illumina sequencing in at least 90% of the progeny (Li and He 2014; Mousavi et al. 2016; Wang et al. 2016; Zhao et al. 2017). We used JoinMap v4.1 (van Ooijen 2006) to calculate linkage relationships and to define linkage groups of RAD markers (supplementary method SM4, Supplementary Material online). Linkage groups were determined using maximum likelihood, and map distances were calculated using Haldane’s map function. Scaffolds were anchored to their appropriate linkage groups using the NCBI BLAST utility to blast linkage group RAD markers against the fragScaff assembly.

### Reconciliation of LACHESIS Assembly to Linkage Map

We used custom Perl scripts to compare the LACHESIS assembly to the linkage map and to reconcile the LACHESIS clusters with the linkage groups. LACHESIS has been shown to be correct at local scales but has produced some large-scale misassemblies (Burton et al. 2013). The RAD linkage map orders scaffolds correctly at large scales, but its resolution is limited by the number of progeny analyzed and hence does not provide as much scaffold orientation information as does LACHESIS. Therefore, we used the LACHESIS orderings for chromosome-scale scaffolding of the *R. williamsianum* genome and the linkage map orderings to correct putative large-scale misassemblies in the LACHESIS results.

### Assessments of Genome Assemblies

We calculated a variety of statistics for the genome assembly of *R. williamsianum* using a modified version of assembly-stats v1.0.1 (Pathogen Informatics 2018). Assembly-stats was used to calculate scaffold number, scaffold sizes, and total bp assembled by ALLPATHS-LG, fragScaff, and in our final assembly.

We assessed the final genomic assembly of *R. williamsianum* with BUSCO v2.0 (Simão et al. 2015) using the embryophyta_odb9 lineage data set representing benchmarking universal single-copy orthologs (BUSCOs, *n* = 1440) from 30 species. BUSCO interacted with AUGUSTUS v3.2.3 (Keller et al. 2011), BLAST+ v2.2.29 (Camacho et al. 2009), and HMMER v3.1b2 (Eddy 2011). Default BUSCO parameters were used under the genome mode, with *Arabidopsis* as the starting species for AUGUSTUS parameters and the AUGUSTUS optimization mode for self-training.

### Repetitive Element Annotation

Repeat elements were annotated and masked in the *R. williamsianum* final genomic assembly using de novo and homology-based methods. We first created a de novo species-specific repeat database for the *R. williamsianum* genome using RepeatModeler v1.0.11 (Smit and Hubley 2008). RepeatModeler uses two de novo repeat finding programs, RECON v1.08 (Bao and Eddy 2002) and RepeatScout v1.05 (Price et al. 2005), along with Tandem Repeats Finder v4.07b (Benson 1999) and NSEG v20000620 (Wootton and Federhen 1993). Three-way degenerative International Union of Biochemistry (IUB) codes within the *R. williamsianum* genomic sequence were replaced with Ns for input into RepeatModeler. Default settings within RepeatModeler were used with the NCBI search engine RMBlast v2.6.0+ (Smit and Hubley 2017).

We then performed repeat annotation and masking with MAKER v2.31.9 (Cantarel et al. 2007; Holt and Yandell 2011) using the de novo species-specific repeat database above and homology-based methods within MAKER. MAKER uses RepeatMasker v4.0.7 (Smit et al. 2017), Tandem Repeats Finder, the RepBase RepeatMasker Edition 20170127 (Jurka et al. 2005; Genetic Information Research Institute 2017), and RepeatRunner (Smith et al. 2007; Yandell 2007). First, we used default settings within RepeatMasker and the NCBI search engine RMBlast v2.6.0+ (Smit and Hubley 2017), in combination with all repeats from RepBase and the de novo species-specific repeat database above, to identify repeats in the *R. williamsianum* genome. Next, we identified transposable elements and viral proteins using the RepeatRunner protein database included in MAKER. Complex repeats were hard-masked, while simple repeats were soft-masked, in the *R. williamsianum* genome for subsequent gene predictions.

We estimated percent repetitive content by first extracting the RepeatMasker and RepeatRunner features from the MAKER annotations and using SOBAcl (Moore 2016) to summarize total base pairs of repetitive elements from MAKER annotations. We then calculated percent repetitive elements from total bp (including runs of 20 Ns or more) assembled in the genome and for each linkage group (LG). Assuming strings of Ns represent repetitive regions as well, we also calculated percent of N-runs in the final genome assembly and for each LG to obtain a grand total estimate of percent repetitive content.

### Structural Annotation

Gene annotation was done within MAKER v2.31.9 (Cantarel et al. 2007; Holt and Yandell 2011) under default settings, except as noted below, using ab initio gene predictions from AUGUSTUS v3.2.3 (Keller et al. 2011). First, we aligned transcriptome data from *Rhododendron delavayi* Franch. (Zhang, Xu, et al. 2017) and proteins from the UniProtKB/Swiss-Prot database 2017_12 release (The UniProt Consortium 2017) and from manually annotated, complete *Actinidia chinensis* Planch. genes (Pilkington et al. 2018) to the *R. williamsianum* masked genome using TBlastX and BlastX from BLAST+ v2.6.0 (Camacho et al. 2009), respectively, within MAKER. Exonerate v2.2.0 (Slater and Birney 2005) was used within MAKER to polish these BLAST alignments. Final gene annotations were produced by MAKER, using the BUSCO training parameters from AUGUSTUS produced during the BUSCO analysis above. Additionally, we rescued genes that were omitted from the default MAKER annotation if they encoded a Pfam (Finn et al. 2016) domain to create a standard build, as outlined by Campbell et al. (2014).

We assessed the completeness of these structural gene annotations for the *R. williamsianum* genome with BUSCO v2.0 (Simão et al. 2015). We used software dependencies as above, the embryophyta_odb9 lineage data set, and default BUSCO parameters under the protein mode, with *Arabidopsis* as the starting species for AUGUSTUS parameters. Additionally, we generated statistics for structural gene annotations using SOBAcl and accessory scripts.

### Functional Annotation

Genes predicted by MAKER in the *R. williamsianum* genome were functionally annotated by Blast2GO (B2G) v4.1.9 and 5.1.12 (Conesa et al. 2005; Götz et al. 2008). Protein sequences from *R. williamsianum* were queried against the UniProtKB/Swiss-Prot database 2017_12 release (The UniProt Consortium 2017) using local BLAST with default settings in B2G but with an expectation value of 1.0E-5, the number of BLAST hits limited to one, and BLAST description annotator enabled. *Rhododendron williamsianum* protein sequences were then compared with protein domain annotations with InterProScan (Jones et al. 2014) through the European Molecular Biology Laboratory-European Bioinformatics Institute (EMBL-EBI) InterPro (Finn et al. 2017) option in B2G. All available families, domains, sites, and repeats, as well as all member databases and other sequence features available in InterPro were used. Gene Ontology (GO) terms from BLAST and InterPro hits were retrieved from the GO database (Ashburner et al. 2000; The Gene Ontology Consortium 2017), selected, and assigned to corresponding *R. williamsianum* query sequences using the B2G default annotation rule (Conesa et al. 2005) and evidence code weights. We also retrieved enzyme code and KEGG (Ogata et al. 1999) pathway map annotations from GO annotations in B2G. Functional GO classes within each of the three GO domains (Biological Process, Cellular Component, Molecular Function) were filtered for classes containing a minimum of 10% of the annotated *R. williamsianum* sequences for each domain. We used REVIGO TreeMaps (Supek et al. 2011) under default settings to summarize GO terms within GO domains into a subset of nonredundant terms based on semantic similarity.

### Comparative Genomics

For comparison of *R. williamsianum* chromosomes to other diploid Ericaceae, *Vaccinium macrocarpon* (cranberry) is available as an assembly with scaffolds anchored to chromosomes (Polashock et al. 2014; Schlautman et al. 2017). We used anchored/ordered scaffolds on LGs to generate pseudochromosomal sequences to identify syntenic relationships both within the *R. williamsianum* genome as well as between *R. williamsianium* and *V. macrocarpon*. For both genomes, these sequences were extracted from the masked genome assembly by stitching anchored/ordered scaffolds from each LG together with 100-N spacers between scaffolds. The coding sequence for pseudochromosomal sequences was then extracted from the MAKER gene predictions. We also examined two other genomes available from Ericaceae, that lack chromosome-level scaffolding, for syntenic relationships: *R. delavayi* (Zhang, Xu, et al. 2017) and *V. corymbosum* L. (blueberry) (Bian et al. 2014; Gupta et al. 2015). For all syntenic analyses, we used SynMap2 within CoGe (Lyons et al. 2008; Haug-Baltzell et al. 2017) with default settings, except for choosing discontinuous MegaBLAST, e-value of 0.0001, as the BLAST algorithm, and 40 genes as the maximum distance between gene pairs in DAGChainer (Haas et al. 2004). Syntenic regions from the DAGChainer output in SynMap2 within the *R. williamsianum* genome and between *R. williamsianum* and *V. macrocarpon* were also visualized with Circos v0.69-6 and 0.69-9 (Krzywinski et al. 2009). Syntenic blocks identified by DAGChainer were converted to bundles using custom scripts and displayed as ribbons in Circos with the chromosome order optimized by the orderchr tool. To calculate syntenic coverage, the total length of syntenic genes along pseudochromosomal sequences from each genome was divided by the total length of predicted genes on pseudochromosomal sequences. We also used SynFind (Tang et al. 2015) within the CoGe platform to estimate syntenic depth among *R. williamsianum* chromosomes as a proxy for the number of whole-genome duplication (WGD) events. We used default settings for SynFind, except for selecting LastZ as the comparison algorithm and a minimum number of five anchoring genes to call a region syntenic.

For identifying WGD in Ericaceae genomes, we used the distribution of synonymous substitutions/site (Ks) between paralogous gene pairs (Lynch and Conery 2000) across the entire genome for *R. delavayi*, *R. williamsianum*, *V. corymbosum*, and *V. macrocarpon*. We used the entire genome to extract Ks estimates to better compare Ks distributions across all species examined because chromosome-scale assemblies were not available for *R. delavayi* and *V. corymbosum.* Additionally, Ks analyses of *R. williamsianum* using syntenic genes from the SynMap2 analyses above produced results (supplementary fig. S9, Supplementary Material online) that were not as clear as analyzing genomic paralogs (paranome), potentially due to fewer data points in the syntenic gene data set. Therefore, we used Ks distributions from all genomic paralogs and compared these to Ks distributions generated for *Actinidia chinensis* by Shi et al. (2010) and in our current study using *A. chinensis* genome data from Pilkington et al. (2018) to validate our methods. We used WGDdetector (Yang et al. 2019), which estimates Ks distributions across gene families to correct for redundant Ks values among paralogs, with MMseqs2 (Steinegger and Söding 2017) as the cluster engine. We then used SiZer (Chaudhuri and Marron 1999) to identify significant peaks in Ks distributions based on significant zero crossings of derivatives from kernel density estimation as in Barker et al. (2008). We used SiZerSM (Marron 2000) in Matlab R2018a (v9.4, The MathWorks, Inc.) with default settings of α = 0.05 and 401 bins. We then fit normal mixture models to the Ks data using maximum likelihood with EMMIX (McLachlan and Peel 1999) as in Barker et al. (2008). We tested one to five components with an unrestricted covariance matrix, 1,000 random starts, and 100 k-means clustering-based starts. Model selection was done by the Bayesian Information Criterion (BIC) within EMMIX. To estimate timing of WGDs, we generated Ks distributions of orthologous gene pairs between *R. williamsianum* and the following genomes using the wgd pipeline v1.0.1 (Zwaenepoel and Van de Peer 2019), which used BLAST v2.7.1 (Altschul et al. 1997) with an e-value cut-off of 1e-5, MCL v14.137 (van Dongen 2000), MUSCLE v3.8.31 (Edgar 2004), and PAML v4.9i (Yang 2007): *A. chinensis* (Pilkington et al. 2018), *Camellia sinensis* (Wei et al. 2018), *V. macrocarpon* (Polashock et al. 2014), and *Vitis vinifera* (The French–Italian Public Consortium for Grapevine Genome Characterization et al. 2007). We then compared these Ks distributions, which represented speciation events, to the Ks distribution of *R. williamsianum* genomic paralogs, which represented WGDs. Histograms and normal mixture models were plotted in R v3.6.1 (R Core Team 2018).

## Results

### De Novo Assembly of *R. williamsianum* Genomic Sequence

In order to produce a de novo assembly of the *R. williamsianum* genome, we first created five *R. williamsianum* genomic libraries with different insert sizes (supplementary table S1, Supplementary Material online) for use with the ALLPATHS-LG assembler (Gnerre et al. 2011; Ribeiro et al. 2012). Total genome sequencing coverage was 388×. We used assembly-stats v1.0.1 (Pathogen Informatics 2018) to calculate scaffold number, scaffold sizes, and total bp assembled by ALLPATHS-LG. The initial assembly yielded 18,269 scaffolds for a total of 491.6 Mb (table 1) out of a genome size of 650.8 Mb as estimated by ALLPATHS-LG. Scaffold sizes ranged with an N10, N50, and N90 of 508,357, 132,014, and 10,942 bp, respectively (table 1). The length of the scaffolds generated by ALLPATHS-LG is limited, probably by the occurrence of dispersed repetitive sequences, such as those belonging to the copia and gypsy families. Therefore, to improve the ALLPATHS-LG assembly, we created a high molecular weight genomic library for contiguity preserving transposase sequencing (CPT-seq) (Amini et al. 2014) and fragScaff scaffolding (Adey et al. 2014) prior to chromosome-scale scaffolding using contact probability maps and genetic linkage. fragScaff was used with the CPT-seq data to conjoin many of the smaller ALLPATHS-LG scaffolds. After assembly with the described parameters that favored conjoining of smaller scaffolds, the final scaffold count was reduced to 11,962, for a total of 532.5 Mb, with an N10, N50, and N90 of 931,684, 225,489, and 29,431 bp, respectively (table 1), corresponding to fold improvements of 1.83, 1.71, and 2.69, respectively. The modest improvements to N10 and N50 in the fragScaff assembly were primarily due to tailoring the algorithm to prioritize smaller scaffold incorporation, thus favoring increase in the N90 as well as significantly (35%) reducing the total number of scaffolds. Using fragScaff after assembly of genomic libraries by ALLPATHS-LG resulted in an improved assembly of the *R. williamsianum* genome through the reduction in scaffold number and increase in scaffold size.

**Table 1:** Combining ALLPATHS-LG with fragScaff Improved the De Novo Assembly of the *Rhododendron williamsianum* Genome

Assembly/Stats	ALLPATHS-LG	fragScaff	Final^a^
No. scaffolds	18,269	11,962	11,985
Scaffold N10 size (bp)	508,357	931,684	815,789
Scaffold N50 size (bp)	132,014	225,489	218,828
Scaffold N90 size (bp)	10,942	29,431	29,444
Total bp	491,643,723	532,499,445	532,123,622

a Combined results from ALLPATHS-LG, fragScaff, and linkage map. Linkage analysis split misassembled scaffolds, resulting in slightly more scaffolds. Scaffolds were also filtered for duplicates and mitochondrial contamination.

### Chromosome-Scale Scaffolding of *R. williamsianum* Genome Using Two Independent Methods

We used a combination of results from a linkage map based on our RAD sequencing data and LACHESIS (Burton et al. 2013) analysis of our Hi-C data to cluster and order the *R. williamsianum* scaffolds along chromosomes. The ordering of *R. williamsianum* scaffolds from the fragScaff assembly within linkage groups (LGs) was facilitated by the ordering of RAD markers (Etter et al. 2011) from a genetic cross of *R*. “Moonstone” (*R. williamsianum*×*R. campylocarpum*)×*R. campylocarpum*. For a scaffold to be assigned to a LG by this method, its sequence must include one of the RAD markers used to analyze this cross. These markers could only be ordered with respect to one another if they were separated by a scoreable crossover during 1 of the 220 meioses contributing to our 110 progeny. A total of 54,432 *PstI* sites occur in the assembled *R. williamsianum* genome, offering the potential of identifying over 100,000 RAD markers. RAD sequencing parents and progeny of the cross allowed selection of 11,616 polymorphic RAD markers with sufficient coverage in the progeny (supplementary fig. S1, Supplementary Material online). Progeny genotypes at these markers were analyzed by JoinMap which designated 13 linkage groups and ordered blocks of completely linked RAD markers along the chromosomes.

This method produced chromosomal segments, each defined by a group of RAD markers inseparable from one another by crossover frequencies, and ordered those blocks of markers along each chromosome. Scaffolds sharing sequences with these marker blocks could then be assigned to the chromosome segments, and scaffolds with marker sites in two or more contiguous blocks could be oriented on their chromosomes. RAD markers used in this study allowed us to place 1,711 scaffolds along chromosomes, for a total of 363.8 Mb, representing 68% of the assembled genome.

The JoinMap linkage analysis uncovered 23 scaffold-assembly errors. We used the Linux program *grep* to identify scaffolds containing RAD markers that did not all map to the same linkage group. Targeting one of the single or multiple *N*’s occurring in each of the presumed error-containing regions between incorrectly conjoined groups of RAD markers, we split misassembled scaffolds and reassigned each of the resulting subscaffolds to its appropriate chromosome. Early in the sequencing/assembly project, we discovered we were unable to PCR amplify regions of ALLPATHS-LG scaffolds which contained a single N, suggesting that a lone N signals misassembly. Therefore, we split misassembled scaffolds at single *N*’s whenever possible. After correcting these assembly errors, the final assembly consisted of 11,985 scaffolds, with a total length of 532.1 Mb, and an updated N10, N50, and N90 of 815,789, 218,828, and 29,444 bp, respectively (table 1).

We then used LACHESIS, which has been shown to be effective at local-scale ordering and orienting of scaffolds (Burton et al. 2013), to map *R. williamsianum* scaffolds to chromosomes. Short-read sequencing of a *R. williamsianum* Hi-C library (Lieberman-Aiden et al. 2009) cataloged the frequency with which DNA sequences became crosslinked to one another inside formalin-treated nuclei, elucidating their relative proximities. The LACHESIS software combined the fragScaff assembled sequences with the Hi-C data to order and orient *R. williamsianum* scaffolds within chromosomes (called “clusters” in LACHESIS; supplementary fig. S2, Supplementary Material online).

**Fig. 1.**
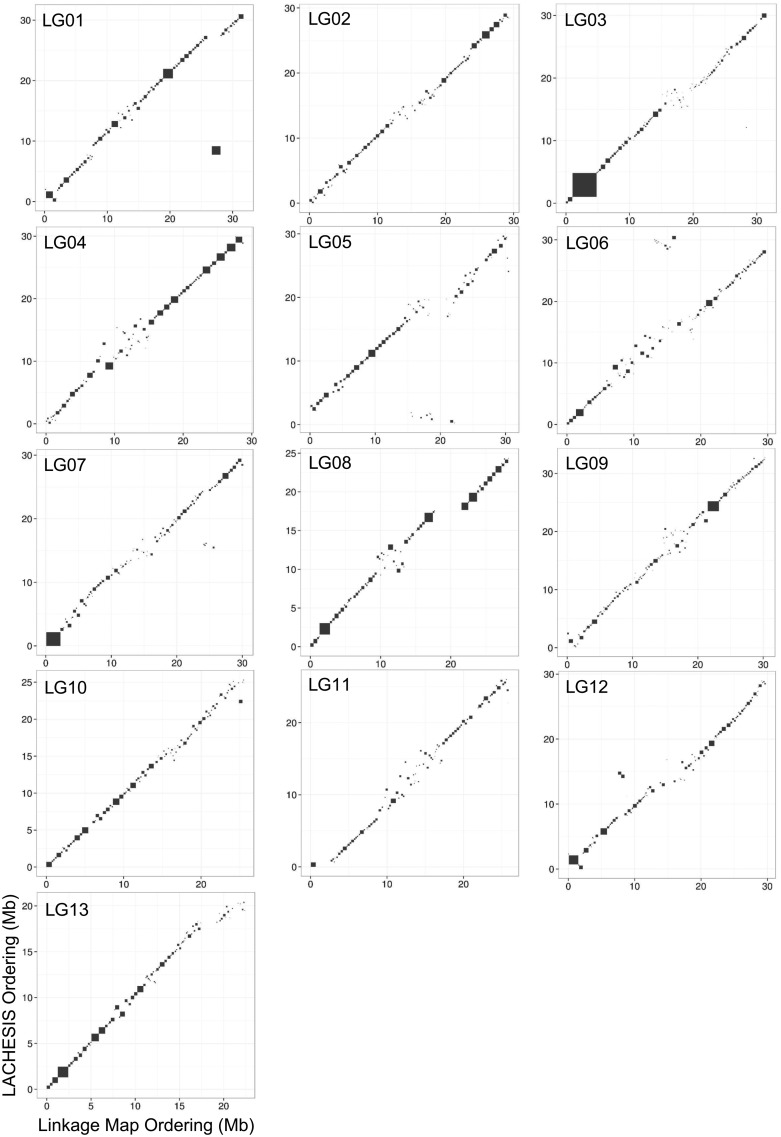
—Comparison of chromosome-scale scaffolding for the *Rhododendron williamsianum* genome by two methods. Comparison of ordering and orienting scaffolds from the *R. williamsianum* de novo assembly within linkage groups based on two methods, LACHESIS assembly of Hi-C data and linkage map of RAD-seq data.

Custom Perl scripts were used to compare the LACHESIS assembly to the linkage map and to identify the JoinMap LG to which each LACHESIS cluster corresponded. Comparison of results obtained by these two methods showed a high degree of concordance, with similar placement of scaffolds in LGs (supplementary fig. S3, Supplementary Material online). For the final chromosomal-scale assembly, the placement and orientation of scaffolds was specified by LACHESIS since LACHESIS provides more orientation data than does the linkage map. However, LACHESIS is known to produce occasional large-scale inversion errors (Burton et al. 2013). Such inversions between the two mapping methods were corrected based on the RAD linkage map (fig. 1 and supplementary fig. S3, Supplementary Material online).

The resulting chromosomal-scale scaffolding has 13 LGs and assigns most of the assembled sequence to LGs (table 2). A total of 3,984 scaffolds with length 394.5 Mb, representing 74% of the assembled genome, were assigned to chromosomes: 1,708 scaffolds (368.4 Mb), representing 69% of the assembled genome, were ordered, and 2,276 scaffolds (26.1 Mb), representing 5% of the assembled genome, were unordered on chromosomes (table 2). The 1,708 clustered and ordered scaffolds included a conservative set of 1,333 scaffolds (327.4 Mb), or 62% of the assembled genome, representing scaffolds ordered by both LACHESIS and the linkage map with no inconsistencies. After our chromosome-scale scaffolding, 8,001 scaffolds (137.7 Mb), representing 26% of the assembled genome, remained unmapped to chromosomes (unclustered in table 2). Combining the local-scale ordering and orientation data provided by the LACHESIS assembly and the large-scale ordering from the linkage map improved the clustering and ordering of scaffolds along chromosomes.

**Table 2:** Chromosome-Scale Scaffolding Statistics for the Final *Rhododendron williamsianum* Genome

Statistics	Final Assembly^a^
Clustered scaffolds	3,984
Total bp clustered	394,456,490
Clustered and ordered scaffolds	1,708
Total bp clustered and ordered	368,385,547
Clustered and unordered scaffolds	2,276
Total bp clustered and unordered	26,070,943
Unclustered scaffolds	8,001
Total bp unclustered	137,667,132
Total scaffolds in final assembly	11,985

a Final assembly used LACHESIS results for clustering and ordering scaffolds and linkage map results for fixing large-scale inversions.

### Assessing Completeness of Final *R. williamsianum* Assembled Genome

We compared the final *R. williamsianum* assembled genome to a conserved set of 1,440 benchmarking universal single-copy ortholog (BUSCO) (Simão et al. 2015) groups across embryophytes to estimate the completeness of the *R. williamsianum* genome. BUSCO analyses estimated the genome to be 89% complete, represented by 89% complete BUSCOs recovered from the genome: 85.2% were single-copy and 3.8% were duplicated. The other 11% BUSCOs not fully recovered from the *R. williamsianum* genome were fragmented (1.9%) or missing (9.1%). Thus, the final assembled genome recovered ∼89% of the *R. williamsianum* genome as determined by single-copy ortholog analyses.

### Annotation of *R. williamsianum* Genome

We identified and masked repetitive elements within the *R. williamsianum* final genome assembly with de novo (RepeatModeler) (Smit and Hubley 2008) and homology-based (RepBase, RepeatRunner) (Jurka et al. 2005; Smith et al. 2007; Yandell 2007) methods using RepeatMasker (Smit et al. 2017) within the MAKER pipeline (Cantarel et al. 2007; Holt and Yandell 2011). From these combined methods, we estimate that 26.4% of the assembled genome consists of identifiable repetitive elements. Additionally, assuming that strings of Ns in our final assembly are repetitive regions adds another 32.4% of repetitive content. Therefore, we estimate that a grand total of 58.8% of the *R. williamsianum* final assembled genome consists of repetitive sequences.

We also used the MAKER pipeline to predict genes within the *R. williamsianum* assembled genome. We obtained estimates of 23,559 genes in the final assembled genome (table 3) using the standard build in MAKER, which has been shown to balance sensitivity and specificity (Campbell et al. 2014). Of these predicted genes, 91% were on ordered scaffolds assigned to LGs, 2% were on unordered scaffolds assigned to LGs, and 7% were on unclustered scaffolds. The mean Annotation Edit Distance (AED) (Holt and Yandell 2011), which measures how well the gene predictions match the evidence for predicted genes, from the standard build was 0.3. Initially, 94% of predicted genes from the default build had an AED ≤0.5. After rescuing genes with Pfam domains, 88% of predicted genes from the standard build had an AED ≤0.5. The total number of predicted genes encompasses on an average 0.044 genes per kb across the assembled genome (table 3). These genes were on an average 4,628 bp long, with 5.68 exons per gene, an exon length of 212 bp, and an intron length of 645 bp (table 3). Taken together, our gene predictions estimate that at least 20% of the assembled *R. williamsianum* genome encodes mRNA and only 5% is actual coding sequence.

**Table 3:** Statistics for the Structural Gene Annotation of the *Rhododendron williamsianum* Genome

Gene Statistic	Final Assembly
Total predicted genes	23,559
Gene density/kb	0.044
Average gene length (bp)	4,628
Average no. exons/gene	5.68
Average exon length (bp)	212
Average intron length (bp)	645

**Fig. 2.**
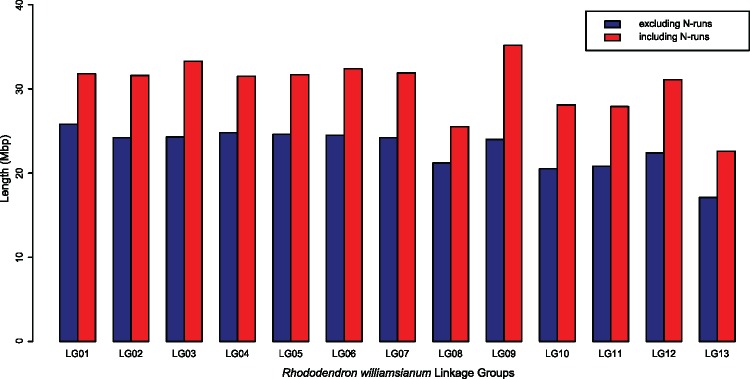
—Estimates of chromosome size for the *Rhododendron williamsianum* genome. Chromosome sizes were estimated by summing the lengths of ordered and unordered scaffolds within each linkage group (LG). Two chromosome size estimates are provided for each LG, including and excluding runs of 20 Ns or more.

We estimated the *R. williamsianum* chromosome sizes by summing up ordered and unordered scaffolds assigned to each LG, either excluding or including runs of 20 Ns or more (fig. 2). Estimated chromosome lengths ranged from 17.1 Mb for LG13 to 25.8 Mb for LG01 when runs of Ns were excluded, or from 22.6 Mb for LG13 to 35.2 Mb for LG09 when runs of Ns were included (fig. 2). We then used the lengths (including runs of 20 Ns or more) to get an idea of percent repeats and percent mRNAs in each chromosomal sequence. We estimate that LGs are on an average 50.7% (±3.80) repetitive and 26.1% (±2.87) mRNAs. We do not see large differences in repeat content or gene content among chromosomes.

We again compared the *R. williamsianum* set of predicted genes to a conserved set of 1,440 BUSCO groups across embryophytes to estimate completeness of the structural gene annotation done within the MAKER pipeline above. BUSCO analyses estimated gene annotation as 79.1% complete, represented by 79.1% complete BUSCOs recovered from the gene predictions: 75.4% were single-copy and 3.7% were duplicated. The other 20.9% BUSCOs not fully recovered from the gene predictions were fragmented (9.2%) or missing (11.7%). Thus, our structural gene annotation using the MAKER pipeline recovered ∼79% of the potential genes in the *R. williamsianum* genome.

**Fig. 3.**
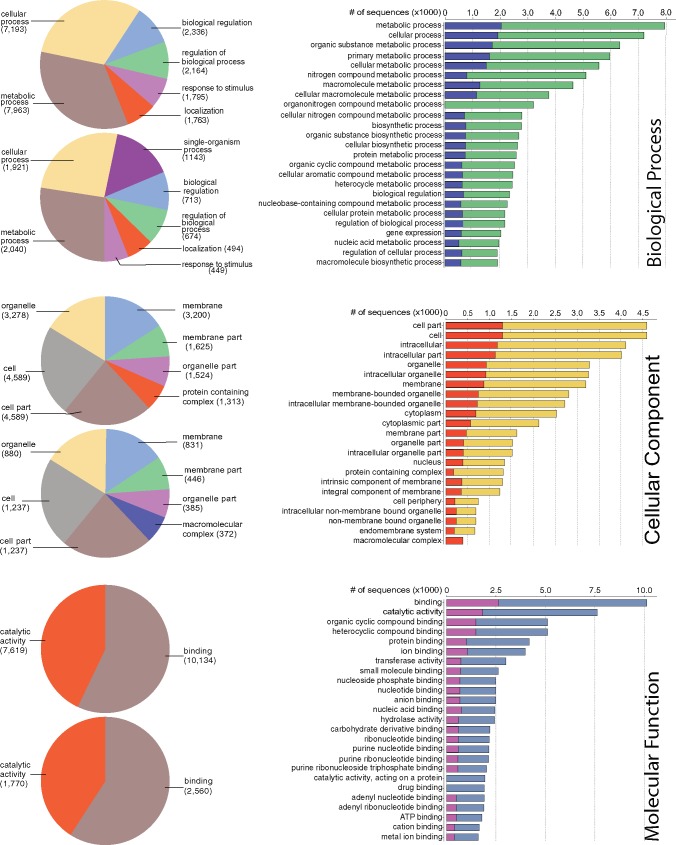
—Gene Ontology (GO) classification of functionally annotated, predicted genes in *Rhododendron williamsianum*. The three panels represent the three main GO domains. Each panel represents level 2 classes from the GO directed acyclic graph (left side) and the top 25 classes across all levels (right side). Left side: top pie chart represents all annotated genes in genome, bottom pie chart represents syntenic genes within genome. Right side: blue bars represent syntenic genes, green bars represent all annotated genes for biological process; orange bars represent syntenic genes, yellow bars represent all annotated genes for cellular component; pink bars represent syntenic genes, blue bars represent all annotated genes for molecular function. Only classes with at least 10% of annotated genes within a domain are listed.

We then used the Blast2GO (B2G) (Conesa et al. 2005; Götz et al. 2008) pipeline to functionally annotate genes predicted by MAKER in the *R. williamsianum* genome. The B2G pipeline functionally classified 18,538 out of 23,559 (79%) predicted genes to Gene Ontology (GO) terms. Of the functionally annotated genes, 62% (11,445 genes) belonged to biological process, 35% (6,462 genes) to cellular component, and 83% (15,472 genes) to molecular function, with overlap among these GO domains. Distribution of genes within each of these three GO domains are shown in figure 3 among GO classes at various levels. For details on functional classes represented in the *R. williamsianum* genome, refer to figure 3 and supplementary table S2, Supplementary Material online.

### Syntenic Relationships among *R. williamsianum* Chromosomes Identify Multiple WGDs

**Fig. 4.**
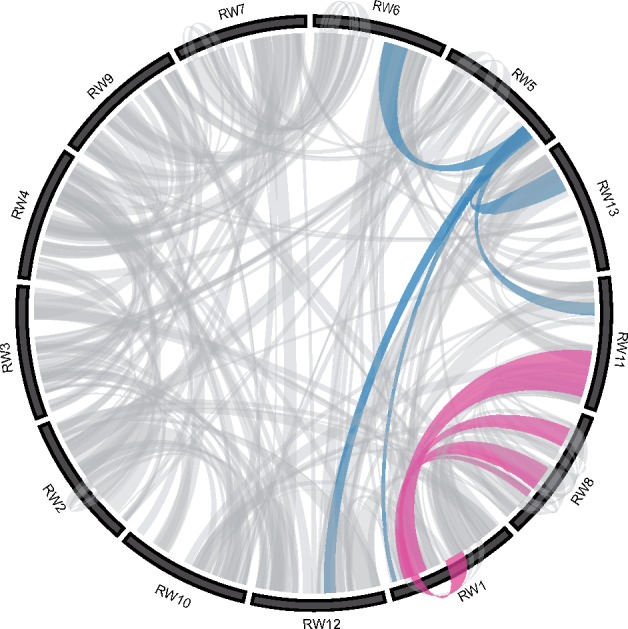
—Syntenic blocks within the *Rhododendron williamsianum* genome indicate multiple whole-genome duplications. The 13 chromosomes of *R. williamsianum* (RW) are arranged along the circumference of the Circos (Krzywinski et al. 2009) plot to reduce crossing of bundles. Each bundle represents a block of at least five syntenic gene pairs shared between two chromosomes (interior bundles) or within a chromosome (exterior bundles). Colored bundles highlight two syntenic regions with 1:5 syntenic depths.

Because of increasing evidence of ancient WGDs in plants (Ren et al. 2018), we used SynMap2 within the Comparative Genomics (CoGe) platform (Lyons et al. 2008; Haug-Baltzell et al. 2017) to search for blocks of syntenic gene pairs between *R. williamsianum* chromosomes, which would indicate evidence of WGD. We found 7,151 syntenic gene pairs across 520 syntenic blocks on ordered scaffolds assigned to chromosomes from the *R. williamsianum* genome (fig. 4 and supplementary fig. S4 and table S3, Supplementary Material online). Of predicted genes on ordered scaffolds (100.8 Mb), 34% (34.0 Mb) were syntenic to genes on other chromosomes within the *R. williamsianum* genome, indicating WGD. We also used SynFind (Tang et al. 2015) within the CoGe platform to estimate syntenic depth among chromosomes as a proxy for the number of paleopolyploid events (supplementary table S4, Supplementary Material online). We found evidence of multiple rounds of WGD within the *R. williamsianum* genome where a given syntenic region has more than one homolog as indicated by syntenic depths ranging from 1:2 to 1:5 (supplementary table S4, Supplementary Material online). For example, the same region on chromosome 5 is homologous to regions on chromosomes 1, 6, 11, 12, and 13, indicating a syntenic depth of 1:5 (fig. 4 and supplementary fig. S4, Supplementary Material online). These syntenic relationships within the *R. williamsianum* genome suggest multiple rounds of WGD in the history of this genome.

In order to determine what genes may be preferentially retained after WGDs in *R. williamsianum*, we extracted GO terms for syntenic genes from our whole-genome B2G functional annotations. We were able to retrieve functional annotations for 6,025 out of 7,151 syntenic genes. Of the functionally annotated syntenic genes, 49% (2,979 genes) belonged to biological process, 28% (1,704 genes) to cellular component, and 62% (3,763 genes) to molecular function, with overlap among these GO domains. Figure 3 shows, for each GO domain, how syntenic genes are distributed among GO classes compared with all genes in the genome. Distributions of syntenic gene functions are similar to the distributions of all gene functions for the most part, but syntenic genes are enriched in certain GO classes compared with the whole-genome (fig. 3). In particular, syntenic genes are enriched for an additional class from level 2 in the GO directed acyclic graph for biological process and cellular component. Under biological process, syntenic genes are enriched in single-organism process, which includes genes involved in multicellular organism development, response to oxidative stress, cell redox homeostasis, and protein phosphorylation (supplementary table S5, Supplementary Material online). Under cellular component, syntenic genes are enriched in macromolecular complex, which includes genes primarily involved in ribosomes (supplementary table S6, Supplementary Material online). Thus, we see certain genes with particular functions as being preferentially retained in duplicate after WGDs in *R. williamsianum*.

### Syntenic Relationships between *Rhododendron* and Cranberry Chromosomes

**Fig. 5.**
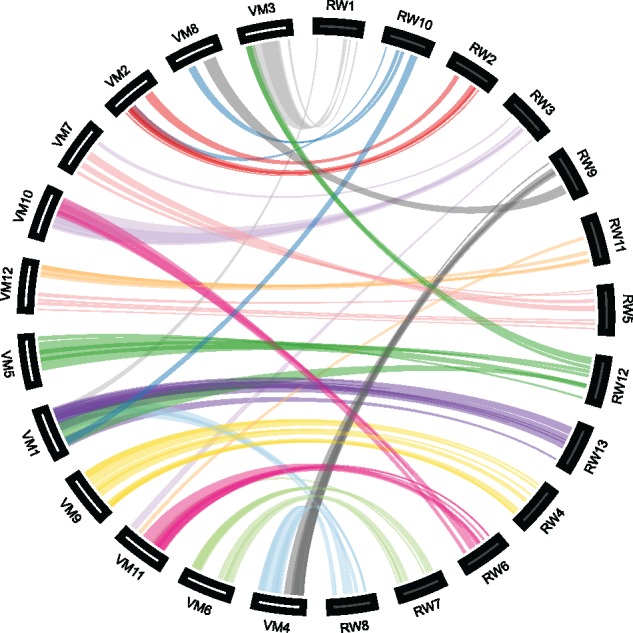
—Syntenic blocks between Ericaceae genomes, *Rhododendron williamsianum* and *Vaccinium macrocarpon* (cranberry). The 12 chromosomes of *V. macrocarpon* (VM) and 13 chromosomes of *R. williamsianum* (RW) are arranged along the circumference of the Circos (Krzywinski et al. 2009) plot to reduce crossing of bundles. Each colored bundle represents a block of at least five syntenic gene pairs shared between two chromosomes.

In order to determine how conserved chromosome organization is within the Ericaceae, we used SynMap2 within the CoGe platform to identify blocks of syntenic gene pairs between the two available diploid Ericaceae genomes with chromosome-scale scaffolding, *R. williamsianum* and *V. macrocarpon* (cranberry) (Polashock et al. 2014; Schlautman et al. 2017). We were only able to extract 2,128 predicted genes from anchored scaffolds from the current publicly available *V. macrocarpon* assembly versus 21,419 predicted genes from *R. williamsianum* ordered scaffolds. However, we did find 526 syntenic gene pairs across 81 syntenic blocks on anchored/ordered scaffolds assigned to chromosomes between the *R. williamsianum* and *V. macrocarpon* genomes (fig. 5 and supplementary fig. and table S7, Supplementary Material online). From these, we can draw certain inferences about the orthology of chromosomes between *R. williamsianum* and *V. macrocarpon*. For example, we see a reciprocal one-to-one relationship between *V. macrocarpon* chromosome 9 and *R. williamsianum* chromosome 4 (fig. 5 and supplementary fig. S5 and table S7, Supplementary Material online). In some cases, *V. macrocarpon* chromosomes appear orthologous to more than one *R. williamsianum* chromosome, suggesting WGD in the *R. williamsianum* genome. For example, the same region on *V. macrocarpon* chromosome 1 is orthologous to *R. williamsianum* chromosomes 8, 12, and 13; on *V. macrocarpon* chromosome 10, the same region is orthologous to *R. williamsianum* chromosomes 3 and 6. In other cases, it is difficult to determine whether the orthology of *V. macrocarpon* chromosomes to more than one *R. williamsianum* chromosome is due to fission or fusion of chromosomes versus WGD followed by gene/chromosome loss. For example, different parts of *V. macrocarpon* chromosome 4 are orthologous to *R. williamsianum* chromosome 8 or 9, similarly with *V. macrocarpon* chromosome 8 to *R. williamsianum* chromosome 9 or 10 and *V. macrocarpon* chromosome 12 to *R. williamsianum* chromosome 5 or 11. Therefore, we sought evidence of WGD in cranberry and other Ericaceae genomes to aid inferences about chromosome orthology.

### Syntenic Relationships within Other Ericaceae Genomes Identify WGDs

We wanted to determine whether the syntenic evidence of WGD in *R. williamsianum* was found in other Ericaceae. Again, we used SynMap2 within the CoGe platform to search for blocks of syntenic gene pairs within the cranberry (*V. macrocarpon*) genome. We did not find any syntenic blocks within the *V. macrocarpon* genome. This could be due to either the lack of WGD or the low number of predicted genes on anchored scaffolds. Two other diploid genomes are available within Ericaceae but do not have chromosome-level scaffolding: *Rhododendron delavayi* and *Vaccinium corymbosum* (blueberry). We used the entire assembly for each of these genomes in SynMap2 to see if there was any signal of WGD in either genome. Syntenic blocks are more dispersed since the scaffolds are not anchored to chromosomes, however, we do find syntenic regions between different scaffolds in both genomes (supplementary figs. S6 and S7, Supplementary Material online), indicating WGD in both *R. delavayi* and *V. corymbosum* genomes.

### Synonymous Substitutions in Ericaceae Genomes Indicate Multiple WGDs

**Fig. 6.**
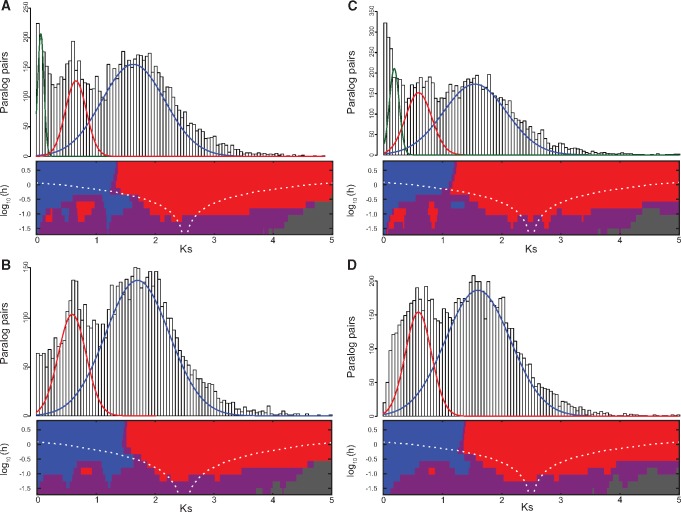
—Distributions of synonymous substitutions/site (Ks) for paralogous gene pairs in Ericaceae genomes. For each genome, top panel shows histogram of Ks data overlaid by normal mixture model from EMMIX (McLachlan and Peel 1999). Two or three components of the normal mixture model are shown in green, red, and blue that correspond with SiZer (Chaudhuri and Marron 1999) results below. Bottom panel shows two or three significant peaks identified by SiZer map, where blue indicates significant increases and red indicates significant decreases in curves; purple is not significant, gray indicates sparse data. (*A*) *Rhododendron delavayi*. (*B*) *R. williamsianum*. (*C*) *Vaccinium corymbosum*. (*D*). *V. macrocarpon*.

In order to estimate the number and relative timing of WGDs evident in Ericaceae genomes, we used the distribution of synonymous substitutions/site (Ks) between paralogous gene pairs across entire genomes available for *R. delavayi*, *R. williamsianum*, *V. corymbosum*, and *V. macrocarpon*, and compared these distributions to Ks distributions generated for *Actinidia chinensis* by Shi et al. (2010) and in our current study (supplementary fig. S8, Supplementary Material online). SiZer (Chaudhuri and Marron 1999) identified two (*R. williamsianum*, *V. macrocarpon*) or three (*R. delavayi*, *V. corymbosum*) significant peaks in Ks distributions, two of which appear shared across all four genomes (red and blue curves, fig. 6). An additional, more recent peak was identified in *R. delavayi* and *V. corymbosum* (green curves, fig. 6), but these taxa are known to be diploid (Janaki Ammal et al. 1950; Bian et al. 2014). Therefore, this third peak likely represents background gene duplication and loss (Blanc and Wolfe 2004) rather than recent WGD. We tested normal mixture models with one to five components in EMMIX (McLachlan and Peel 1999) to estimate parameters for Ks distributions for each genome. Models selected by Bayesian Information Criterion in EMMIX included four or five components for each of the four genomes, but we only show those components that match SiZer results (fig. 6). The ages of two Ks peaks identified in each *Rhododendron* genome correspond to one another (fig. 6*A* and *B*) and indicate two potential shared WGDs that were likely inherited from a common ancestor of *Rhododendron*. The mean Ks values for the two peaks in *R. delavayi* are 0.67 and 1.65; the mean Ks values for the two peaks in *R. williamsianum* are 0.61 and 1.71 (table 4). Similarly, the ages of two Ks peaks identified in each *Vaccinium* genome correspond to one another (fig. 6*C* and *D*) and indicate two potential shared WGDs that were likely inherited from a common ancestor of *Vaccinium*. The mean Ks values for the two peaks in *V. corymbosum* are 0.59 and 1.54; the mean Ks values for the two peaks in *V. macrocarpon* are 0.60 and 1.60 (table 4). Because lineages are likely to have different rates of molecular evolution, we would expect estimates of Ks from a shared WGD to be slightly different among Ericales genomes. However, these two peaks across all four Ericaceae genomes correspond well to two WGDs identified in *Actinidia chinensis* (Shi et al. 2010) and in our current study (mean Ks 0.49 and 1.72; table 4); we refer to them as *Ad*-β and *At*-γ, respectively, as denoted by Shi et al. (2010). To verify whether the two shared WGDs, we found across Ericaceae genomes represent the *Ad*-β and *At*-γ WGD events, we generated Ks distributions of orthologous gene pairs between *R. williamsianum* and the following genomes: *A. chinensis* (Pilkington et al. 2018), *Camellia sinensis* (Wei et al. 2018), *V. macrocarpon* (Polashock et al. 2014), and *Vitis vinifera* (The French–Italian Public Consortium for Grapevine Genome Characterization et al. 2007). We then compared these Ks distributions, which represent speciation events, to the two WGDs in Ericaceae as represented in *R. williamsianum* (supplementary fig. S10, Supplementary Material online). Based on these comparisons, we found that the most recent WGD in *R. williamsianum* (red star, supplementary fig. S10, Supplementary Material online) likely occurred before the speciation events between *R. williamsianum* and Ericales relatives *Actinidia, Camellia*, and *Vaccinium*, and after the speciation event between *R. williamsianum* and *Vitis*, corresponding to the timeline for *Ad*-β. The oldest WGD in *R. williamsianum* (blue star, supplementary fig. S10, Supplementary Material online) occurred before all four speciation events above, corresponding to the timeline for *At*-γ. Therefore, we propose that the evidence of two shared WGDs from Ks data in *Rhododendron* and *Vaccinium* genomes represents two ancient, shared WGDs that originated in a common ancestor of Ericaceae and even further back in a common ancestor in the Ericales.

**Table 4:** Normal Mixture Model Parameters Estimated by EMMIX for Synonymous Substitutions/Site (Ks) Distributions of Ericales Genomes

Genome	*Ad-*β Mean Ks	*At-*γ Mean Ks
*Actinidia chinensis*	0.49	1.72
*Rhododendron delavayi*	0.67	1.65
*Rhododendron williamsianum*	0.61	1.71
*Vaccinium corymbosum*	0.59	1.54
*Vaccinium macrocarpon*	0.60	1.60

## Discussion

The experiments, data, and conclusions we present here provide a detailed view of the genomes of *R. williamsianum* and other ericaceous species. We used Illumina short sequencing reads to assemble scaffolds which allowed us to ascertain ab initio the sets of contiguous DNA sequences that comprise the *R. williamsianum* nuclear genome. We then determined the chromosomal distribution and order of these sequences by LACHESIS assembly of Hi-C data and linkage analysis of RAD-seq data. We annotated the *R. williamsianum* genome and used predicted genes to identify syntenic blocks of genes within this genome, which illuminated multiple syntenic relationships across chromosomes. Using Ks distributions from *R. williamsianum* and three other publicly available diploid genomes in the heath family, we find shared evidence of at least two ancient WGDs across these members of the Ericaceae.

Our ALLPATHS-LG estimate for the genome size of *R. williamsianum* (651 Mb) is slightly less than the estimate from the other genome sequenced in subg. *Hymenanthes*, *R. delavayi* (696 Mb) (Zhang, Xu, et al. 2017), and less than the minimum genome size reported for the subgenus based on flow cytometry (694 Mb) (Jones et al. 2007). This could reflect a slightly smaller genome size for *R. williamsianum* than those of other subg. *Hymenanthes* rhododendrons. In any event, knowledge of the clustering and ordering of DNA sequences for this species will be a useful genomic resource for other rhododendrons. Using the same (BUSCO) method of assessment for genome completeness, *R. williamsianum* is 89% and *R. delavayi* is 93% complete (Zhang, Xu, et al. 2017). Thus, two largely complete *Rhododendron* genome assemblies are now publicly available.

We identified transposable elements (TEs) representing 26% of the *R. williamsianum* assembly, which is lower than the 40% identified as TEs in the *V. macrocarpon* genome (Polashock et al. 2014). Both estimates above were produced by the MAKER pipeline and are lower than estimates in the *R. delavayi* genome using a custom pipeline (52%) (Zhang, Xu, et al. 2017). However, when we include strings of Ns as potentially repetitive regions, the *R. williamsianum* total repetitive content (59%) is comparable to *R. delavayi*. Similarly, gene annotation for the *R. delavayi* genome is 87% complete using a custom pipeline, but 78% using the MAKER pipeline (Zhang, Xu, et al. 2017), which may indicate that custom pipelines can be more exhaustive in their predictions than the MAKER pipeline.

Our MAKER pipeline gene annotation of the *R. williamsianum* genome is comparable (79% complete) to the *R. delavayi* MAKER annotation above. More genes (32,938) were predicted for the *R. delavayi* 695-Mb assembly using the custom pipeline versus the 23,559 genes predicted for the 532-Mb *R. williamsianum* assembly (table 3). However, gene density is comparable for the two genomes with 0.047/kb predicted for *R. delavayi* (Zhang, Xu, et al. 2017) and 0.044/kb predicted for *R. williamsianum*. For *R. delavayi*, mean statistics for gene length (=4434 bp), exons/gene (=4.62), exon length (=250 bp), and intron length (=785 bp) (Zhang, Xu, et al. 2017) were close to the mean gene length (=4628 bp), exons/gene (=5.68), exon length (=212 bp), and intron length (=645 bp) of *R. williamsianum* (table 3). Gene-density predictions for these *Rhododendron* genomes are notably different from those for *Vaccinium* genomes, which have two to four times more predicted genes per kb of sequence. *Vaccinium macrocarpon* has a predicted gene density of 0.083/kb (Polashock et al. 2014), and *V. corymbosum* has a predicted gene density of 0.162/kb (Gupta et al. 2015), suggesting that *Vaccinium* genomes are more gene-rich than *Rhododendron* genomes.

For functional annotation comparisons between available *Rhododendron* subg. *Hymenanthes* genomes, 86% of predicted genes in *R. delavayi* (Zhang, Xu, et al. 2017) and 79% of predicted genes in *R. williamsianum* were functionally annotated. These functional annotations are comparable to the functional annotation of a recently developed transcriptome from mixed tissues for *R. latoucheae* Franch. (Xing et al. 2017) from subg. *Choniastrum* (Franchet) Drude, with 81% of unigenes functionally annotated. For GO classification, the percentage of genes assigned to molecular function in *R. williamsianum* is higher than that reported for *R. latoucheae* (Xing et al. 2017), otherwise, biological process terms are more dominant than cellular component terms in both genomes. Within each of these three GO domains, the top two dominant classes are the same for *R. latoucheae* and *R. williamsianum* (fig. 3). The similarity in abundance of GO classes of functional genes across these two different subgenera, *Hymenanthes* and *Choniastrum*, implies conservation in functional sequence across the genus.

The LACHESIS technique maps sequences based on their intranuclear proximities, while linkage mapping is based upon meiotic recombination frequencies. Because the two methods rely on conceptually divergent data generation techniques and analyses, the congruence of their assignment and ordering of sequences on chromosomes (fig. 1 and supplementary fig. S3, Supplementary Material online) reinforces these conclusions. The few discrepancies between the LACHESIS assembly and linkage map were primarily inversions of multiscaffold blocks (fig. 1 and supplementary fig. S3, Supplementary Material online), which may be due to problematic regions in the *R. williamsianum* assembly, such as the regions around centromeres or those that may have resulted from segmental duplications or other types of repeats. Similar ordering and orientation errors have been seen in the human LACHESIS assembly (Burton et al. 2013). After corrections to rectify large-scale inversions (fig. 1 and supplementary fig. S3, Supplementary Material online), discrepancies between our LACHESIS and linkage map results mostly occur near the center of chromosomes which may indicate that most of the *R. williamsianum* chromosomes are metacentric (fig. 1). Metacentric chromosomes have also been observed in other Ericaceae members such as *Vaccinium macrocarpon* (Schlautman et al. 2017).

We have generated a linkage map for *R. williamsianum*, which used 11,616 markers from RAD-seq data, in combination with the LACHESIS assembly, to cluster and order 1,708 scaffolds (table 2), representing 368.4 Mb (69%) of the 532 Mb assembly and 21,419 predicted genes. Two other linkage map studies have been published for *Rhododendron*, from subg. *Hymenanthes* and *Tsutsusi*, using 332 to 523 combined AFLP, EST, MYB, RAPD, RFLP, and/or SSR markers, with the goal of identifying genes involved in flower color, iron-chlorosis tolerance, leaf color, and leaf morphology for cultivation purposes (Dunemann et al. 1999; De Keyser et al. 2010, 2013). These studies identified 13, 16, or 18 linkage groups, despite the known haploid chromosome number of 13 for the genus (Sax 1930). The *R. williamsianum* linkage map generated in our study provides a higher density of markers and confirms a haploid chromosome number of 13.

Within subgenus *Hymenanthes*, interspecies crosses are fertile, yielding stable hybrid progeny (Ma et al. 2010). These, in turn, prove to be fertile in further crosses with subg. *Hymenanthes* species or hybrids. On this basis, we would expect the genome sequence of *R. williamsianum*, presented here, to share synteny with other *Rhododendron* species in subg. *Hymenanthes*, but this remains to be tested with the acquisition of other *Rhododendron* genomes with chromosome-scale assemblies. In contrast, crosses that involve species in different subgenera give hybrids inefficiently or not at all. One exception is the case of azaleas in subg. *Pentanthera* crossed with elepidote rhododendrons in subg. *Hymenanthes*, which can yield sterile hybrids (Kehr 1977). Among the possible reasons for this lack of interfertility is a fundamental difference in gene arrangement between the subgenera. As genome structures are ascertained for other *Rhododendron* subgenera, molecular karyotyping will enable reconciliation of genome structure with taxonomy.

*Rhododendron* and *Vaccinium* genomes used in our study are known to be diploid (Janaki Ammal et al. 1950; Bian et al. 2014; Polashock et al. 2014), and had no prior evidence of ancient WGD in these genomes. Despite the availability of scaffolds anchored to chromosomes, it was previously unknown whether *V. macrocarpon* had experienced any past WGDs (Schlautman et al. 2015). With our chromosome-level assembly of the *R. williamsianum* genome, we find spatial evidence of WGDs in syntenic analyses within this genome. In our analyses of genome assemblies for other Ericaceae, available without chromosome-level mapping, we also find spatial (syntenic) and temporal (Ks) evidence of WGDs across all Ericaceae genomes, highlighting at least two shared, ancient WGDs.

**Fig. 7.**
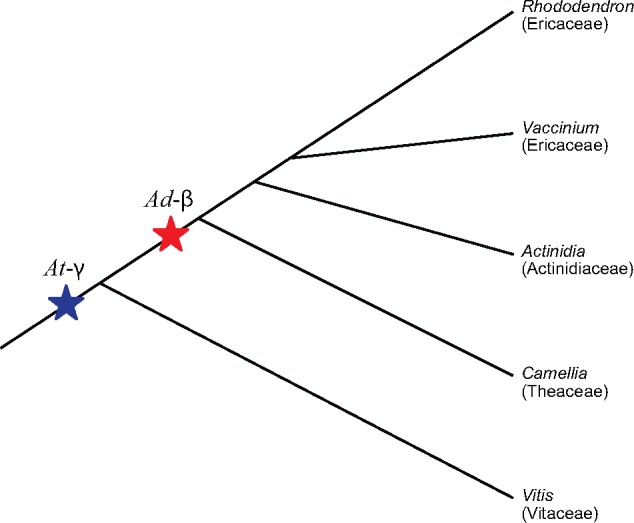
—Whole-genome duplication events detected in Ericaceae genomes. Phylogeny of Ericales genomes sequenced to date and outgroup *Vitis vinifera*. Whole-genome duplication (WGD) events detected in Ericaceae genomes in this study indicated by blue and red stars. *At*-γ is named after WGD detected in *Arabidopsis thaliana* (Bowers et al. 2003); *Ad*-β is named after WGD detected in *Actinidia* (Shi et al. 2010).

Previous studies in other genomes from the order Ericales have found two shared, ancient WGDs in both *Actinidia* (Actinidiaceae) and *Camellia* (Theaceae) (Shi et al. 2010; Huang et al. 2013; Xia et al. 2017; Wei et al. 2018), with Ks estimates comparable to our Ericaceae estimates (table 4). The oldest duplication (*At-γ*) dates back to an ancient hexaploidy shared by most eudicots ∼120 Ma (Jiao et al. 2012; Vekemans et al. 2012). The second duplication (*Ad-β*) is shared in a common ancestor of *Actinidia*+*Camellia*, estimated somewhere between 64.3 and 101.4 Ma (Shi et al. 2010; Huang et al. 2013; Xia et al. 2017; Wei et al. 2018). Two WGDs in *Rhododendron* and *Vaccinium* appear to result from the same events because we find evidence for these two WGDs arising at similar times across both genera and before their divergence (fig. 6 and supplementary fig. S10, Supplementary Material online). These WGDs must have been present in a common ancestor of the two groups, which is dated to ∼77 Ma (Rose et al. 2018). We also show that the most recent WGD arose before the speciation event between *Rhododendron* and its Ericales relatives (*Actinidia* and *Camellia*) and the oldest WGD arose before the speciation event between *Rhododendron* and *Vitis*. Therefore, we propose that the two WGDs we find in ericaceous genomes each represent one of the products of WGD as found in *Actinidia* and *Camellia* (fig. 7); the most recent of these resulting from a WGD in a common ancestor of Actinidiaceae+Ericaceae+Theaceae (*Ad-β*), and the oldest representing a hexaploid event in a eudicot ancestor (*At-γ*), as evidenced by syntenic depths of up to 1:5 in *R. williamsianum*. Supporting this is a recent study by Landis et al. (2018) who used Ks data from the 1KP project (Matasci et al. 2014) to identify and date WGDs across angiosperms and found evidence for two shared, ancient WGDs in Ericaceae transcriptomes: the oldest dating to 105.7 Ma and the second to 85.6 Ma in a common ancestor in the Ericales.

The *R. williamsianum* genome presented here adds to the growing body of genomic resources available for the genus. One other nuclear genome within the genus has been sequenced (Zhang, Xu, et al. 2017), and at least nine transcriptomes have been recently published (Fang et al. 2017; Xing et al. 2017; Zhang, Zhang, et al. 2017; Cheng et al. 2018; Choudhary et al. 2018; Li et al. 2018; Wang et al. 2018; Xiao et al. 2018; Zhao et al. 2018). We hope that the availability of chromosome-scale scaffolding for the *R. williamsianum* genome will motivate other efforts in the genus and across the heath family to provide similar resources for examining chromosome evolution across these groups. Our study also highlights that future genomic sequencing endeavors with chromosome-scale scaffolding should include syntenic analyses within the genome of interest before cross-species comparisons, as there may be signals of ancient WGDs in these genomes.

## Supplementary Material

Supplementary data are available at *Genome Biology and Evolution* online.

## Supplementary Material

evz245_Supplementary_Data

## Notes

**Data deposition:** DNA sequencing data and final genome assembly for *Rhododendron williamsianum* have been deposited at NCBI BioProject under the accession PRJNA432092 and at CoGe under genome ID 51679. In the genome assembly, within each linkage group, all ordered scaffolds are given in their LACHESIS order, followed by all unordered scaffolds listed in descending order of length. Unclustered scaffolds are listed after linkage group 13 in descending order of length. The scripts used for genome assembly, annotation, and comparative genomics are available in the GitHub repository (https://github.com/vsoza/rhododendron-genome/). Synteny analyses within the Comparative Genomics (CoGe) platform can be obtained from the links given. *Rhododendron delavayi*: https://genomevolution.org/r/14kzh (whole-genome); *Rhododendron williamsianum*: https://genomevolution.org/r/12blb, https://genomevolution.org/r/17qc6 (ordered scaffolds); *Vaccinum corymbosum*: https://genomevolution.org/r/14oui. (whole-genome); *Vaccinium macrocarpon*: https://genomevolution.org/r/14kz6 (anchored scaffolds), https://genomevolution.org/r/14m7b (whole-genome); and *R. williamsianum* and *V. macrocarpon*: https://genomevolution.org/r/152k5 (anchored/ordered scaffolds).

## References

[evz245-B1] Adey A, et al 2014 In vitro, long-range sequence information for de novo genome assembly via transposase contiguity. Genome Res. 24(12):2041–2049. 25327137 10.1101/gr.178319.114 PMC4248320

[evz245-B2] Altschul SF, et al 1997 Gapped BLAST and PSI-BLAST: a new generation of protein database search programs. Nucleic Acids Res. 25(17):3389–3402. 9254694 10.1093/nar/25.17.3389 PMC146917

[evz245-B3] Altschul SF, Gish W, Miller W, Myers EW, Lipman DJ. 1990 Basic local alignment search tool. J Mol Biol. 215(3):403–410. 2231712 10.1016/S0022-2836(05)80360-2

[evz245-B4] Amini S, et al 2014 Haplotype-resolved whole-genome sequencing by contiguity-preserving transposition and combinatorial indexing. Nat Genet. 46(12):1343–1349. 25326703 10.1038/ng.3119 PMC4409979

[evz245-B5] Ashburner M, et al 2000 Gene Ontology: tool for the unification of biology. Nat Genet. 25(1):25. 10802651 10.1038/75556 PMC3037419

[evz245-B6] Bao Z, Eddy SR. 2002 Automated de novo identification of repeat sequence families in sequenced genomes. Genome Res. 12(8):1269–1276. 12176934 10.1101/gr.88502 PMC186642

[evz245-B7] Barker MS, et al 2008 Multiple paleopolyploidizations during the evolution of the Compositae reveal parallel patterns of duplicate gene retention after millions of years. Mol Biol Evol. 25(11):2445–2455. 18728074 10.1093/molbev/msn187 PMC2727391

[evz245-B8] Benson G. 1999 Tandem Repeats Finder: a program to analyze DNA sequences. Nucleic Acids Res. 27(2):573–580. 9862982 10.1093/nar/27.2.573 PMC148217

[evz245-B9] Bian Y, et al 2014 Patterns of simple sequence repeats in cultivated blueberries (*Vaccinium* section *Cyanococcus* spp.) and their use in revealing genetic diversity and population structure. Mol Breeding. 34(2):675–689.

[evz245-B10] Blanc G, Wolfe KH. 2004 Widespread paleopolyploidy in model plant species inferred from age distributions of duplicate genes. Plant Cell 16(7):1667–1678. 15208399 10.1105/tpc.021345 PMC514152

[evz245-B11] Bowers JE, Chapman BA, Rong JK, Paterson AH. 2003 Unravelling angiosperm genome evolution by phylogenetic analysis of chromosomal duplication events. Nature 422(6930):433–438. 12660784 10.1038/nature01521

[evz245-B12] Burton JN, et al 2013 Chromosome-scale scaffolding of *de novo* genome assemblies based on chromatin interactions. Nat Biotechnol. 31(12):1119–1125. 24185095 10.1038/nbt.2727 PMC4117202

[evz245-B13] Camacho C, et al 2009 BLAST+: architecture and applications. BMC Bioinformatics 10(1):421. 20003500 10.1186/1471-2105-10-421 PMC2803857

[evz245-B14] Campbell MS, et al 2014 MAKER-P: a tool kit for the rapid creation, management, and quality control of plant genome annotations. Plant Physiol. 164(2):513. 24306534 10.1104/pp.113.230144 PMC3912085

[evz245-B15] Cantarel BL, et al 2007 MAKER: an easy-to-use annotation pipeline designed for emerging model organism genomes. Genome Res. 18(1):188–196. 18025269 10.1101/gr.6743907 PMC2134774

[evz245-B16] Catchen J, Hohenlohe PA, Bassham S, Amores A, Cresko WA. 2013 Stacks: an analysis tool set for population genomics. Mol Ecol. 22(11):3124–3140. 23701397 10.1111/mec.12354 PMC3936987

[evz245-B17] Catchen JM, Amores A, Hohenlohe P, Cresko W, Postlethwait JH. 2011 *Stacks*: building and genotyping loci *de novo* from short-read sequences. G3 (Bethesda) 1:171–182. 22384329 10.1534/g3.111.000240 PMC3276136

[evz245-B18] Chamberlain D. 1996 The genus *Rhododendron*: it’s classification and synonymy. Kew (United Kingdom): Royal Botanic Gardens.

[evz245-B19] Chamberlain DF. 1982 A revision of *Rhododendron*. II. subgenus *Hymenanthes*. Notes R Bot Gard Edinb. 39:209–486.

[evz245-B20] Chaudhuri P, Marron JS. 1999 SiZer for exploration of structures in curves. J Am Stat Assoc. 94(447):807–823.

[evz245-B21] Cheng S, et al 2018 De novo assembly and characterization of *Rhododendron hybridum* hort. (Ericaceae) global transcriptome using Illumina sequencing. Pak J Bot. 50:757–761.

[evz245-B22] Choudhary S, et al 2018 Transcriptome characterization and screening of molecular markers in ecologically important Himalayan species (*Rhododendron arboreum*). Genome 61(6):417–428. 29658317 10.1139/gen-2017-0143

[evz245-B23] Conesa A, et al 2005 Blast2GO: a universal tool for annotation, visualization and analysis in functional genomics research. Bioinformatics 21(18):3674–3676. 16081474 10.1093/bioinformatics/bti610

[evz245-B24] De Keyser E, Lootens P, Van Bockstaele E, De Riek J. 2013 Image analysis for QTL mapping of flower colour and leaf characteristics in pot azalea (*Rhododendron simsii* hybrids). Euphytica 189(3):445–460.

[evz245-B25] De Keyser E, Shu QY, Van Bockstaele E, De Riek J. 2010 Multipoint-likelihood maximization mapping on 4 segregating populations to achieve an integrated framework map for QTL analysis in pot azalea (*Rhododendron simsii* hybrids). BMC Mol Biol. 11(1):1. 20070894 10.1186/1471-2199-11-1 PMC2837023

[evz245-B26] Dunemann F, Kahnau R, Stange I. 1999 Analysis of complex leaf and flower characters in *Rhododendron* using a molecular linkage map. Theor Appl Genet. 98(6–7):1146–1155.

[evz245-B27] Eddy SR. 2011 Accelerated profile HMM searches. PLoS Comput Biol. 7(10):e1002195. 22039361 10.1371/journal.pcbi.1002195 PMC3197634

[evz245-B28] Edgar R. 2004 MUSCLE: multiple sequence alignment with high accuracy and high throughput. Nucleic Acids Res. 32(5):1792–1797. 15034147 10.1093/nar/gkh340 PMC390337

[evz245-B29] Etter PD, Bassham S, Hohenlohe PA, Johnson EA, Cresko WA. 2011 SNP discovery and genotyping for evolutionary genetics using RAD sequencing. Methods Mol Biol. 772:157–178. 22065437 10.1007/978-1-61779-228-1_9 PMC3658458

[evz245-B30] Fang L, et al 2017 *De novo* RNA sequencing transcriptome of *Rhododendron obtusum* identified the early heat response genes involved in the transcriptional regulation of photosynthesis. PLoS One 12(10):e0186376. 29059200 10.1371/journal.pone.0186376 PMC5653301

[evz245-B31] Finn RD, et al 2016 The Pfam protein families database: towards a more sustainable future. Nucleic Acids Res. 44(D1):D279–D285. 26673716 10.1093/nar/gkv1344 PMC4702930

[evz245-B32] Finn RD, et al 2017 InterPro in 2017—beyond protein family and domain annotations. Nucleic Acids Res. 45(D1):D190–D199. 27899635 10.1093/nar/gkw1107 PMC5210578

[evz245-B33] Genetic Information Research Institute. 2017 Repbase-derived RepeatMasker libraries. Mountain View (CA): Genetic Information Research Institute [cited 2017 Feb 1]. Available from: http://www.girinst.org/server/RepBase/; last accessed November 15, 2019.

[evz245-B34] Gnerre S, et al 2011 High-quality draft assemblies of mammalian genomes from massively parallel sequence data. Proc Natl Acad Sci U S A. 108(4):1513–1518. 21187386 10.1073/pnas.1017351108 PMC3029755

[evz245-B35] Götz S, et al 2008 High-throughput functional annotation and data mining with the Blast2GO suite. Nucleic Acids Res. 36(10):3420–3435. 18445632 10.1093/nar/gkn176 PMC2425479

[evz245-B36] Gupta V, et al 2015 RNA-Seq analysis and annotation of a draft blueberry genome assembly identifies candidate genes involved in fruit ripening, biosynthesis of bioactive compounds, and stage-specific alternative splicing. GigaScience 4(1):5. 25830017 10.1186/s13742-015-0046-9 PMC4379747

[evz245-B37] Haas BJ, Delcher AL, Wortman JR, Salzberg SL. 2004 DAGchainer: a tool for mining segmental genome duplications and synteny. Bioinformatics 20(18):3643–3646. 15247098 10.1093/bioinformatics/bth397

[evz245-B38] Hall R. 1998 The plate tectonics of Cenozoic SE Asia and the distribution of land and sea In: Hall R,, Holloway JD, editors. Biogeography and geological evolution of SE Asia. Leiden (Netherlands): Backhuys Publishers p. 99–131.

[evz245-B39] Haug-Baltzell A, Stephens SA, Davey S, Scheidegger CE, Lyons E. 2017 SynMap2 and SynMap3D: web-based whole-genome synteny browsers. Bioinformatics 33(14):2197–2198. 28334338 10.1093/bioinformatics/btx144

[evz245-B40] Holt C, Yandell M. 2011 MAKER2: an annotation pipeline and genome-database management tool for second-generation genome projects. BMC Bioinformatics 12(1):491. 22192575 10.1186/1471-2105-12-491 PMC3280279

[evz245-B41] Huang S, et al 2013 Draft genome of the kiwifruit *Actinidia chinensis*. Nat Commun. 4(1):2640. 24136039 10.1038/ncomms3640 PMC4089393

[evz245-B42] Irving E, Hebda R. 1993 Concerning the origin and distribution of rhododendrons. J Am Rhododendr Soc. 47:139–162.

[evz245-B43] Janaki Ammal EK, Enoch IC, Bridgwater M. 1950 Chromosome numbers in species of *Rhododendron*. Rhododendr Year Book. 5:78–91.

[evz245-B44] Jiao Y, et al 2012 A genome triplication associated with early diversification of the core eudicots. Genome Biol. 13(1):R3. 22280555 10.1186/gb-2012-13-1-r3 PMC3334584

[evz245-B45] Jones JR, Ranney TG, Lynch NP, Krebs SL. 2007 Ploidy levels and relative genome sizes of diverse species, hybrids, and cultivars of *Rhododendron*. J Am Rhododendr Soc. 61:220–227.

[evz245-B46] Jones P, et al 2014 InterProScan 5: genome-scale protein function classification. Bioinformatics 30(9):1236–1240. 24451626 10.1093/bioinformatics/btu031 PMC3998142

[evz245-B47] Jurka J, et al 2005 Repbase update, a database of eukaryotic repetitive elements. Cytogenet Genome Res. 110(1-4):462–467. 16093699 10.1159/000084979

[evz245-B48] Kehr AE. 1977 Azaleodendron breeding. Q Bull Am Rhododendr Soc. 31:226–232.

[evz245-B49] Keller O, Kollmar M, Stanke M, Waack S. 2011 A novel hybrid gene prediction method employing protein multiple sequence alignments. Bioinformatics 27(6):757–763. 21216780 10.1093/bioinformatics/btr010

[evz245-B50] Krzywinski M, et al 2009 Circos: an information aesthetic for comparative genomics. Genome Res. 19(9):1639–1645. 19541911 10.1101/gr.092759.109 PMC2752132

[evz245-B51] Landis JB, et al 2018 Impact of whole-genome duplication events on diversification rates in angiosperms. Am J Bot. 105(3):348–363. 29719043 10.1002/ajb2.1060

[evz245-B52] Leslie AC. 2004 The International Rhododendron Register and Checklist 2nd ed : London: The Royal Horticultural Society Available from: https://www.rhs.org.uk/plants/plantsmanship/plant-registration/Rhododendron-cultivar-registration/Rhododendron; last accessed November 15, 2019.

[evz245-B53] Leslie AC. 2017 The International Rhododendron Register and Checklist (2004): Consolidated Supplement 2003–2017. London: The Royal Horticultural Society. Available from: https://www.rhs.org.uk/plants/plantsmanship/plant-registration/Rhododendron-cultivar-registration/Rhododendron; last accessed November 15, 2019.

[evz245-B54] Li H. 2011 A statistical framework for SNP calling, mutation discovery, association mapping and population genetical parameter estimation from sequencing data. Bioinformatics 27:2987–2993. 21903627 10.1093/bioinformatics/btr509 PMC3198575

[evz245-B55] Li T, et al 2018 Development of novel EST-SSR markers for *Rhododendron longipedicellatum* (Ericaceae) and cross-amplification in two congeners. Appl Plant Sci. 6(6):e01162. 30131904 10.1002/aps3.1162 PMC6025811

[evz245-B56] Li Y, He M. 2014 Genetic mapping and QTL analysis of growth-related traits in *Pinctada fucata* using restriction-site associated DNA sequencing. PLoS One 9(11):e111707. 25369421 10.1371/journal.pone.0111707 PMC4219768

[evz245-B57] Lieberman-Aiden E, et al 2009 Comprehensive mapping of long-range interactions reveals folding principles of the human genome. Science 326(5950):289–293. 19815776 10.1126/science.1181369 PMC2858594

[evz245-B58] Lynch M, Conery JS. 2000 The evolutionary fate and consequences of duplicate genes. Science 290(5494):1151–1155. 11073452 10.1126/science.290.5494.1151

[evz245-B59] Lyons E, Pedersen B, Kane J, Freeling M. 2008 The value of nonmodel genomes and an example using SynMap within CoGe to dissect the hexaploidy that predates the Rosids. Tropical Plant Biol. 1(3-4):181–190.

[evz245-B60] Ma Y, Milne RI, Zhang C, Yang J. 2010 Unusual patterns of hybridization involving a narrow endemic *Rhododendron* species (Ericaceae) in Yunnan, China. Am J Bot. 97(10):1749–1757. 21616807 10.3732/ajb.1000018

[evz245-B61] Marron JS. 2000 SizerSM. [cited 2018 Sep 24] Available from: http://www.unc.edu/∼marron/marron_software.html; last accessed September 24, 2018.

[evz245-B62] Matasci N, et al 2014 Data access for the 1,000 Plants (1KP) project. GigaScience 3(1):2047–217X-3-17. 10.1186/2047-217X-3-17 PMC4306014 25625010

[evz245-B63] McLachlan G, Peel D. 1999 The EMMIX algorithm for the fitting of normal and t-components. J Stat Softw Artic. 4:1–14.

[evz245-B64] Meyer M, Kircher M. 2010 Illumina sequencing library preparation for highly multiplexed target capture and sequencing. Cold Spring Harb Protoc. 2010:pdb.prot5448. 10.1101/pdb.prot5448 20516186

[evz245-B65] Moore B. 2016 SOBA: sequence ontology bioinformatics analysis. [cited 2018 Feb 15] Available from: https://github.com/The-Sequence-Ontology/SOBA; last accessed November 15, 2019.

[evz245-B66] Mousavi M, et al 2016 De novo SNP discovery and genetic linkage mapping in poplar using restriction site associated DNA and whole-genome sequencing technologies. BMC Genomics 17(1):656–656. 27538483 10.1186/s12864-016-3003-9 PMC4991039

[evz245-B67] Ogata H, et al 1999 KEGG: Kyoto encyclopedia of genes and genomes. Nucleic Acids Res. 27(1):29–34. 9847135 10.1093/nar/27.1.29 PMC148090

[evz245-B68] Pathogen Informatics. 2018 assembly-stats. Hinxton, Cambridgeshire (United Kingdom): Pathogen Informatics, Wellcome Sanger Institute. [cited 2018 Jan 23] Available from: https://github.com/sanger-pathogens/assembly-stats; last accessed November 15, 2019.

[evz245-B69] Pilkington SM, et al 2018 A manually annotated *Actinidia chinensis* var. *chinensis* (kiwifruit) genome highlights the challenges associated with draft genomes and gene prediction in plants. BMC Genomics 19(1):257. 29661190 10.1186/s12864-018-4656-3 PMC5902842

[evz245-B70] Polashock J, et al 2014 The American cranberry: first insights into the whole genome of a species adapted to bog habitat. BMC Plant Biol. 14(1):165. 24927653 10.1186/1471-2229-14-165 PMC4076063

[evz245-B71] Price AL, Jones NC, Pevzner PA. 2005 *De novo* identification of repeat families in large genomes. Bioinformatics 21(Suppl 1):i351–i358. 15961478 10.1093/bioinformatics/bti1018

[evz245-B72] R Core Team. 2018 R: A language and environment for statistical computing. Vienna (Austria): R Foundation for Statistical Computing. [cited 2018 Oct 4] Available from: https://www.R-project.org; last accessed November 15, 2019.

[evz245-B73] Ren R, et al 2018 Widespread whole genome duplications contribute to genome complexity and species diversity in angiosperms. Mol Plant. 11(3):414–428. 29317285 10.1016/j.molp.2018.01.002

[evz245-B74] Ribeiro FJ, et al 2012 Finished bacterial genomes from shotgun sequence data. Genome Res. 22(11):2270–2277. 22829535 10.1101/gr.141515.112 PMC3483556

[evz245-B75] Rose JP, et al 2018 Phylogeny, historical biogeography, and diversification of angiosperm order Ericales suggest ancient Neotropical and East Asian connections. Mol Phylogenet Evol. 122:59–79. 29410353 10.1016/j.ympev.2018.01.014

[evz245-B76] Sax K. 1930 Chromosome stability in the genus *Rhododendron*. Am J Bot. 17(4):247–251.

[evz245-B77] Schlautman B, et al 2015 Development of a high-density cranberry SSR linkage map for comparative genetic analysis and trait detection. Mol Breed. 35:177.

[evz245-B78] Schlautman B, et al 2017 Construction of a high-density American cranberry (*Vaccinium macrocarpon* Ait.) composite map using genotyping-by-sequencing for multi-pedigree linkage mapping. G3 (Bethesda) 7:1177–1189. 28250016 10.1534/g3.116.037556 PMC5386866

[evz245-B79] Schwery O, et al 2015 As old as the mountains: the radiations of the Ericaceae. New Phytol. 207(2):355–367. 25530223 10.1111/nph.13234

[evz245-B80] Shi T, Huang H, Barker MS. 2010 Ancient genome duplications during the evolution of kiwifruit (*Actinidia*) and related Ericales. Ann Bot. 106(3):497–504. 20576738 10.1093/aob/mcq129 PMC2924827

[evz245-B81] Simão FA, Waterhouse RM, Ioannidis P, Kriventseva EV, Zdobnov EM. 2015 BUSCO: assessing genome assembly and annotation completeness with single-copy orthologs. Bioinformatics 31(19):3210–3212. 26059717 10.1093/bioinformatics/btv351

[evz245-B82] Slater GSC, Birney E. 2005 Automated generation of heuristics for biological sequence comparison. BMC Bioinformatics 6(1):31. 15713233 10.1186/1471-2105-6-31 PMC553969

[evz245-B83] Sleumer HO. 1980 A system of the genus *Rhododendron* L. In: Contributions toward a classification of *Rhododendron*: Proceedings, International Rhododendron Conference, the New York Botanical Garden, 1978 May 15–17. Bronx (NY): The New York Botanical Garden. p. 1–18.

[evz245-B84] Smit A, Hubley R. 2017 RMBlast. Seattle, WA: Institute for Systems Biology. [cited 2017 Dec 15] Available from: http://www.repeatmasker.org/RMBlast.html; last accessed November 15, 2019.

[evz245-B85] Smit AFA, Hubley R. 2008 RepeatModeler Open-1.0. Seattle, WA: Institute for Systems Biology. [cited 2017 Dec 15] Available from: http://www.repeatmasker.org/RepeatModeler/; last accessed November 15, 2019.

[evz245-B86] Smit AFA, Hubley R, Green P. 2017 RepeatMasker Open-4.0. [cited 2017 Dec 15] Available from: http://www.repeatmasker.org; last accessed November 15, 2019.

[evz245-B87] Smith CD, et al 2007 Improved repeat identification and masking in Dipterans. Gene 389(1):1–9. 17137733 10.1016/j.gene.2006.09.011 PMC1945102

[evz245-B88] Steinegger M, Söding J. 2017 MMseqs2 enables sensitive protein sequence searching for the analysis of massive data sets. Nat Biotechnol. 35(11):1026. 29035372 10.1038/nbt.3988

[evz245-B89] Supek F, Bošnjak M, Škunca N, Šmuc T. 2011 REVIGO summarizes and visualizes long lists of Gene Ontology terms. PLoS One 6(7):e21800. 21789182 10.1371/journal.pone.0021800 PMC3138752

[evz245-B90] Tang H, et al 2015 SynFind: compiling syntenic regions across any set of genomes on demand. Genome Biol Evol. 7(12):3286–3298. 26560340 10.1093/gbe/evv219 PMC4700967

[evz245-B91] The French–Italian Public Consortium for Grapevine Genome Characterization et al. 2007 The grapevine genome sequence suggests ancestral hexaploidization in major angiosperm phyla. Nature 449:463–467. 17721507 10.1038/nature06148

[evz245-B92] The Gene Ontology Consortium. 2017 Expansion of the Gene Ontology knowledgebase and resources. Nucleic Acids Res. 45:D331–D338. 27899567 10.1093/nar/gkw1108 PMC5210579

[evz245-B93] The UniProt Consortium. 2017 UniProt: the universal protein knowledgebase. Nucleic Acids Res. 45:D158–D169. 27899622 10.1093/nar/gkw1099 PMC5210571

[evz245-B94] van Dongen SM. 2000. Graph clustering by flow simulation [Ph.D. thesis]. [Utrecht (Netherlands)]: University of Utrecht.

[evz245-B95] van Ooijen JW. 2006 JoinMap^®^ 4, software for the calculation of genetic linkage maps in experimental populations. Wageningen (Netherlands): Kyazma B.V.

[evz245-B96] Vekemans D, et al 2012 Gamma paleohexaploidy in the stem lineage of core eudicots: significance for MADS-box gene and species diversification. Mol Biol Evol. 29(12):3793–3806. 22821009 10.1093/molbev/mss183

[evz245-B97] Wang J, Li L, Zhang G. 2016 A high-density SNP genetic linkage map and QTL analysis of growth-related traits in a hybrid family of oysters (*Crassostrea gigas × Crassostrea angulata*) using genotyping-by-sequencing. G3 (Bethesda) 6:1417. 26994291 10.1534/g3.116.026971 PMC4856092

[evz245-B98] Wang S, et al 2018 Transcriptome analysis and identification of genes associated with flower development in *Rhododendron pulchrum* Sweet (Ericaceae). Gene 679:108–118. 30176315 10.1016/j.gene.2018.08.083

[evz245-B99] Wei C, et al 2018 Draft genome sequence of *Camellia sinensis* var. *sinensis* provides insights into the evolution of the tea genome and tea quality. Proc Natl Acad Sci U S A. 115(18):E4151. 29678829 10.1073/pnas.1719622115 PMC5939082

[evz245-B100] Wootton JC, Federhen S. 1993 Statistics of local complexity in amino acid sequences and sequence databases. Comput Chem. 17(2):149–163.

[evz245-B101] Xia E-H, et al 2017 The tea tree genome provides insights into tea flavor and independent evolution of caffeine biosynthesis. Mol Plant. 10(6):866–877. 28473262 10.1016/j.molp.2017.04.002

[evz245-B102] Xiao Z, et al 2018 De novo transcriptome analysis of *Rhododendron molle* G. Don flowers by Illumina sequencing. Genes Genomics. 40(6):591–601. 29892944 10.1007/s13258-018-0662-8

[evz245-B103] Xing W, et al 2017 De novo assembly of transcriptome from *Rhododendron latoucheae* Franch. using Illumina sequencing and development of new EST-SSR markers for genetic diversity analysis in *Rhododendron*. Tree Genet Genomes. 13:53.

[evz245-B104] Yandell M. 2007 RepeatRunner. [cited 2017 Dec 15] Available from: http://www.yandell-lab.org/software/repeatrunner.html; last accessed November 15, 2019.

[evz245-B105] Yang Y, Li Y, Chen Q, Sun Y, Lu Z. 2019 WGDdetector: a pipeline for detecting whole genome duplication events using the genome or transcriptome annotations. BMC Bioinformatics 20(1):75. 30760221 10.1186/s12859-019-2670-3 PMC6375192

[evz245-B106] Yang Z. 2007 PAML 4: phylogenetic analysis by maximum likelihood. Mol Biol Evol. 24(8):1586–1591. 17483113 10.1093/molbev/msm088

[evz245-B107] Zhang H-B, Zhao X, Ding X, Paterson AH, Wing RA. 1995 Preparation of megabase-size DNA from plant nuclei. Plant J. 7(1):175–184.

[evz245-B108] Zhang L, Xu P, et al 2017 The draft genome assembly of *Rhododendron delavayi* Franch. var. *delavayi*. GigaScience 6(10):1–11. 10.1093/gigascience/gix076 PMC5632301 29020749

[evz245-B109] Zhang Y, Zhang X, Wang Y-H, Shen S-K. 2017 *De novo* assembly of transcriptome and development of novel EST-SSR markers in *Rhododendron rex* Lévl. through Illumina sequencing. Front Plant Sci. 8:1664. 29018469 10.3389/fpls.2017.01664 PMC5622969

[evz245-B110] Zhao Y, et al 2017 High-density genetic linkage map construction and quantitative trait locus mapping for hawthorn (*Crataegus pinnatifida* Bunge). Sci Rep. 7(1):5492. 28710433 10.1038/s41598-017-05756-5 PMC5511184

[evz245-B111] Zhao Y, et al 2018 Physiological and transcriptomic analysis revealed the involvement of crucial factors in heat stress response of *Rhododendron hainanense*. Gene 660:109–119. 29604462 10.1016/j.gene.2018.03.082

[evz245-B112] Zwaenepoel A, Van de Peer Y. 2019 wgd—simple command line tools for the analysis of ancient whole-genome duplications. Bioinformatics 35(12):2153–2155. 30398564 10.1093/bioinformatics/bty915 PMC6581438

