# Supplementary material for: The Rhododendron Genome and Chromosomal Organization Provide Insight into Shared Whole-Genome Duplications across the Heath Family (Ericaceae)
Source: Genome Biol Evol. 2019 Nov 18;11(12):3353–71. doi: 10.1093/gbe/evz245 (PMC6907397; doi:10.1093/gbe/evz245)
Supplement: evz245_Supplementary_Data [file evz245_supplementary_data.zip › RwillMS_SupplMethods_GBE.pdf]

SUPPLEMENTAL METHODS

SM1) Nuclei were isolated from *Rhododendron williamsianum* flower buds using the method of Zhang et al. (1995) with minor variations. Buds were collected, kept on ice and processed the same day. Twenty to thirty grams of floral tissue (100-150 buds) were rinsed several times in tap water then in deionized water, blotted dry, and blended in 200 ml ice-cold Homogenization Buffer (HB) + β-mercaptoethanol for 30 seconds in an Osterizer 10-speed blender on the puree setting. After filtering through two layers of Miracloth, the filtrate was kept on ice and the tissue retained in the Miracloth was blended again for 30 seconds in another 200 ml ice-cold HB + β-mercaptoethanol. This was done two more times, blending for one minute each time, for a total of four filtrate samples. Filtrate volumes were adjusted to 200 ml and Triton X-100 (20%) was added to each filtrate to a concentration of 0.5%, mixed and incubated on ice for exactly 20 minutes to disrupt chloroplasts before spinning. To avoid disrupting nuclei, Triton X-100 was left out of the subsequent Wash Buffer. Samples were not filtered through Miracloth a second time before washing steps. After the final wash, the samples were pooled. This and subsequent steps used an automatic pipetter with a wide-bore tip. DNA for the short fragment library and the three short jumping libraries was extracted from nuclei from this stage of purification using the DNeasy Plant Mini Kit (Qiagen).

Before extraction of the high molecular weight DNA needed for constructing the long jumping fosmid library and CPT-seq library, nuclei were further purified on a one-step Percoll gradient by pelleting at 1800 xg for 15 minutes in a swinging bucket rotor and re-suspending in 0.5-1.0 ml of Floating Buffer (45 g Percoll + 6 g 5X Isolation Buffer [4.28 g Sucrose + 5 ml 10X HB brought to 10 ml with deionized water]). The resuspended sample was split into two 50-ml centrifuge tubes and half of the remaining Floating Buffer was added to each tube. Tubes were gently mixed and centrifuged at 2500 xg for 10 minutes in a swinging bucket rotor. Purified nuclei were removed from the top of the Floating Buffer, pooled, and washed two times in 1X HB. The resulting pellet was immediately extracted for DNA using the Puregene Kit (Qiagen).

For *de novo* assembly of the genomic sequence, reads were formatted as required using the PrepareAllPathsInputs.pl script included with ALLPATHS-LG (Gnerre et al. 2011; Ribeiro et al. 2012). The reads were assembled with the following parameters (the rest being default): HAPLOIDIFY = TRUE, MAX_MEMORY_GB = 400, and THREADS = 20. Insert size estimates (mean and standard deviation) for each library are specified in Supplemental Table S1. Simultaneous querying of the different libraries for sequence overlaps allowed ALLPATHS-LG to infer the respective positions of the sequenced fragments in the *R. williamsianum* genome.

Data from CPT-seq were assembled by fragScaff v140324.1 (Adey et al. 2014; Amini et al. 2014) using the default options with the following exceptions: -E 4000 (end node size in base pairs), -g 30000 (max size of the smallest in a pair of contigs to make the join), -j 2.25 (favors linking of smaller contigs), -u 2.5 (increased stringency in making scaffolds).

SM2) Approximately $4x10^7$ fresh Percoll-purified nuclei, isolated from *R. williamsianum* by methods described in Zhang et al. (1995) and Supplemental Method SM1, were crosslinked by resuspending the nuclei pellet in 30 ml 10mM Tris-HCl, pH 8.0, 0.44 M sucrose, and 5 mM β-mercaptoethanol at room temperature. To this, 0.94 ml 37% formaldehyde was added and gently mixed prior to incubation. The crosslinking reaction was quenched by adding 1.7 ml of 2.5 M glycine. The reaction was centrifuged at 4,000 xg for 15 minutes, and the supernatant was decanted from the pellet. The pellet was kept frozen at -70°C until lysis was performed. We used Protease Inhibitor Cocktail for plant cell and tissue extracts (Sigma-Aldrich) for the “Cell lysis and chromatin digestion” step.

Heuristic parameters for LACHESIS (Burton et al, 2013) included CLUSTER_N=13, CLUSTER_MIN_RE_SITES=20, CLUSTER_MAX_LINK_DENSITY=1, CLUSTER_NONINFORMATIVE_RATIO=4, ORDER_MIN_N_RES_IN_TRUNK=10, and ORDER_MIN_N_RES_IN_SHREDS=10.

SM3) Fresh plant leaves were rinsed with water and blotted on lint-free paper. We weighed 50-100 mg of leaf tissue, and finely chopped it using a new razor blade for each sample. Chopped plant tissue was placed into a 2-ml Lysing Matrix A tube (MP Biomedical) containing 600 ul of pre-warmed (65°C) Qiagen AP1 buffer + 6 ul RNase A (100 mg/ml). Tubes were placed into a Fast-Prep®-24 benchtop homogenizer (MP Biomedical), and homogenized by processing three times for 20 seconds at 4 meters/second, resting 5 minutes between homogenization steps. Samples were incubated at 65°C for 1 hour, followed by the addition of 195 ul of Qiagen buffer P3. Tubes were incubated on ice for 5 minutes, then centrifuged twice for 5 minutes at 15,000 rpm, moving the supernatant to a Qiashredder column after each spin. After centrifuging the Qiashredder columns, 600 ul of the eluate was moved to a fresh microfuge tube. The extraction protocol was finished according to the Qiagen manufacturer's protocol.

SM4) We used 400 ng of DNA per sample as starting material, and all reaction components were proportionally reduced. Genomic DNA was digested for 4 hours with *PstI* (New England Biolabs), followed by the ligation of barcoded P1 adapters using T4 DNA ligase (New England Biolabs) for 4 hours at ambient temperature. After pooling and size selection, the P2 adapter was attached in a similar manner to the P1 ligation in an overnight reaction. PCR enrichment of the library was carried out in multiple replicates in order to minimize individual reaction bias. The MinElute Gel Purification Kit (Qiagen) was used to elute the final size-selected fragments from an agarose gel. Ampure XP beads (Beckman Coulter) were used in all other recovery steps using a 1:1 ratio of beads to sample in order to eliminate P1 adapter dimers.

RAD sequencing data was processed in Stacks v0.997 (Catchen et al. 2011, 2013) using process_radtags.pl -r -c –q options. Stacks denovo_map.pl was used with the parameters -m 5 -P 2 -M 2 -N 2 -n 0 -t.

For linkage map analysis, JoinMap v4.1(van Ooijen 2006) was used with Independence LOD scores of 5.0-13.0 and a maximum recombination value of 0.40.

References

Adey A et al. 2014. In vitro, long-range sequence information for de novo genome assembly via transposase contiguity. Genome Res. 24:2041–2049. doi: 10.1101/gr.178319.114.

Amini S et al. 2014. Haplotype-resolved whole-genome sequencing by contiguity-preserving transposition and combinatorial indexing. Nat. Genet. 46:1343–1349. doi: 10.1038/ng.3119.

Catchen J, Hohenlohe PA, Bassham S, Amores A, Cresko WA. 2013. Stacks: an analysis tool set for population genomics. Mol. Ecol. 22:3124–3140. doi: 10.1111/mec.12354.

Catchen JM, Amores A, Hohenlohe P, Cresko W, Postlethwait JH. 2011. *Stacks*: building and genotyping loci *de novo* from short-read sequences. G3 Genes Genomes Genet. 1:171–182. doi: 10.1534/g3.111.000240.

Gnerre S et al. 2011. High-quality draft assemblies of mammalian genomes from massively parallel sequence data. Proc. Natl. Acad. Sci. U. S. A. 108:1513–1518. doi: 10.1073/pnas.1017351108.

van Ooijen JW. 2006. *JoinMap® 4, software for the calculation of genetic linkage maps in experimental populations*. Kyazma B.V.: Wageningen, Gelderland, Netherlands.

Ribeiro FJ et al. 2012. Finished bacterial genomes from shotgun sequence data. Genome Res. 22:2270–2277. doi: 10.1101/gr.141515.112.

Zhang H-B, Zhao X, Ding X, Paterson AH, Wing RA. 1995. Preparation of megabase-size DNA from plant nuclei. Plant J. 7:175–184. doi: 10.1046/j.1365-313X.1995.07010175.x.
