# Supplementary material for: The Rhododendron Genome and Chromosomal Organization Provide Insight into Shared Whole-Genome Duplications across the Heath Family (Ericaceae)
Source: Genome Biol Evol. 2019 Nov 18;11(12):3353–71. doi: 10.1093/gbe/evz245 (PMC6907397; doi:10.1093/gbe/evz245)
Supplement: evz245_Supplementary_Data [file evz245_supplementary_data.zip › Supplemental_Figures_GBE_revision_111419_untracked.pdf]

Supplemental Fig. S1. RAD-seq markers used at 11,616 polymorphic sites to create a linkage map of the *Rhododendron williamsianum* genome.

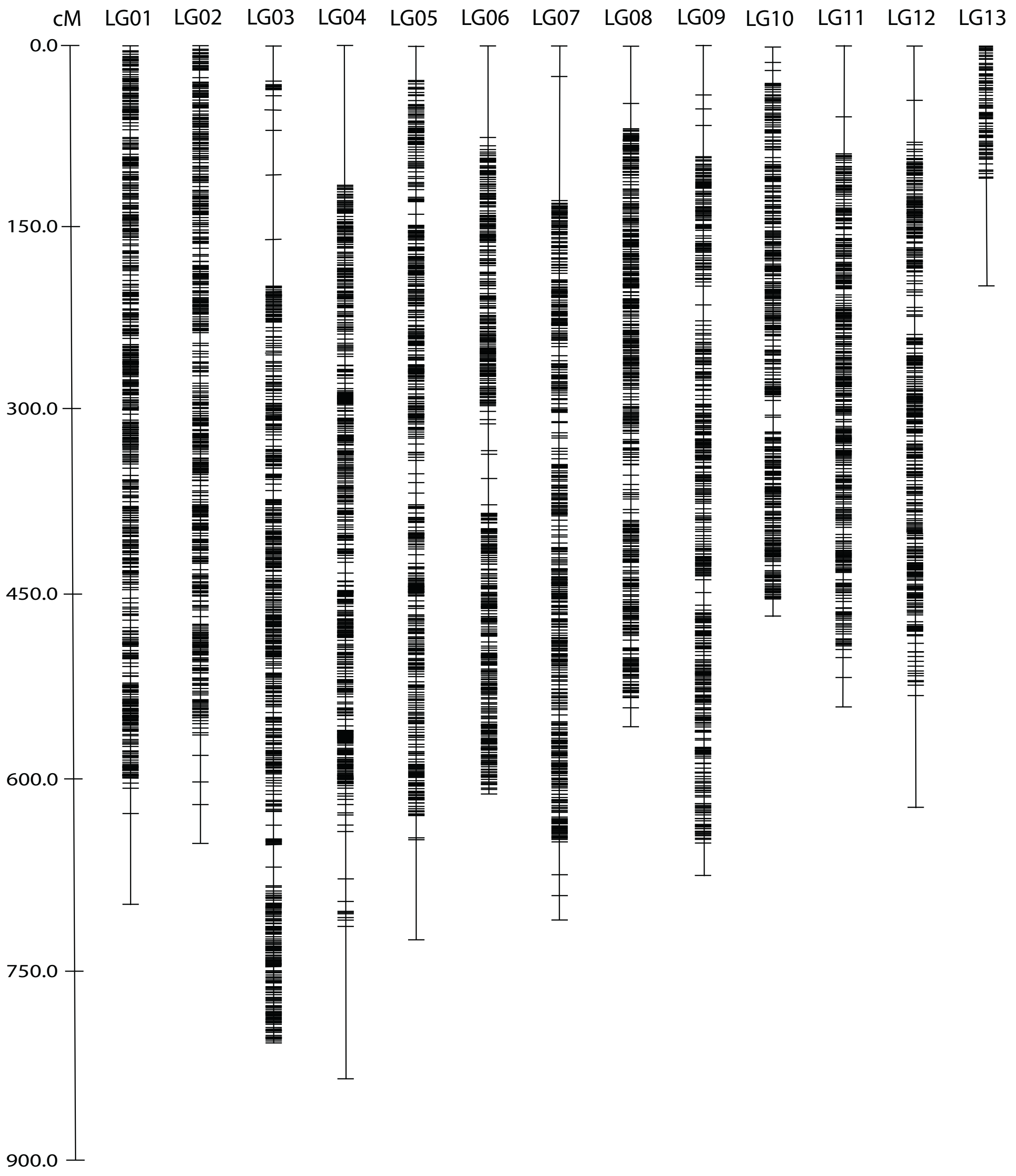

Supplemental Fig. S2. Heatmap of the normalized Hi-C link density between scaffolds across the LACHESIS assembly of the *Rhododendron williamsianum* genome. Scaffolds are represented by rows and columns in heatmap, which are clustered and ordered by LACHESIS. Color intensity corresponds to density of interactions, represented by the total number of links between scaffolds.

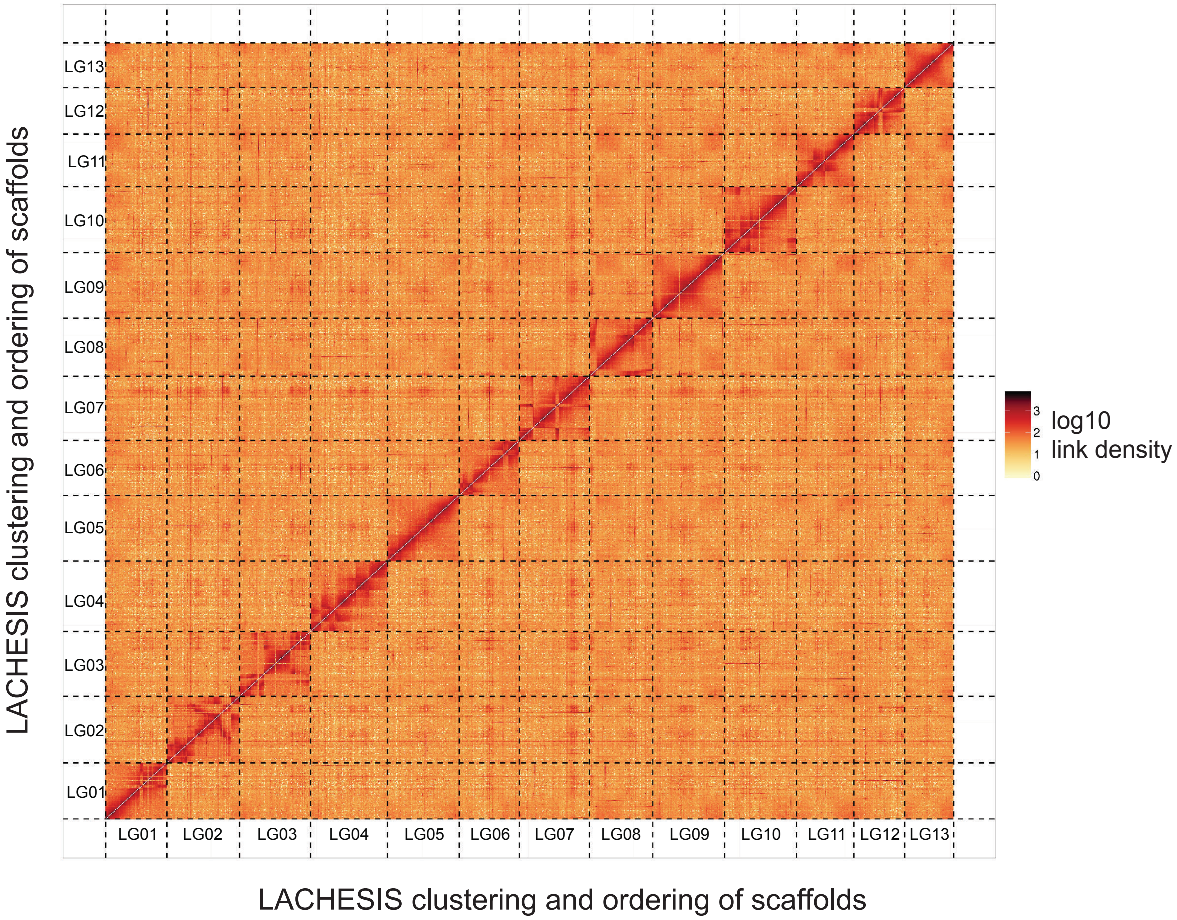

Supplemental Fig. S3. Comparison of ordering and orienting scaffolds from the *Rhododendron williamsianum de novo* assembly within linkage groups based on two methods, LACHESIS assembly of Hi-C data and linkage map of RAD-seq data. For each linkage group, left panel shows initial LACHESIS assembly, right panel shows LACHESIS assembly after adjustments from linkage map to correct large-scale inversions.

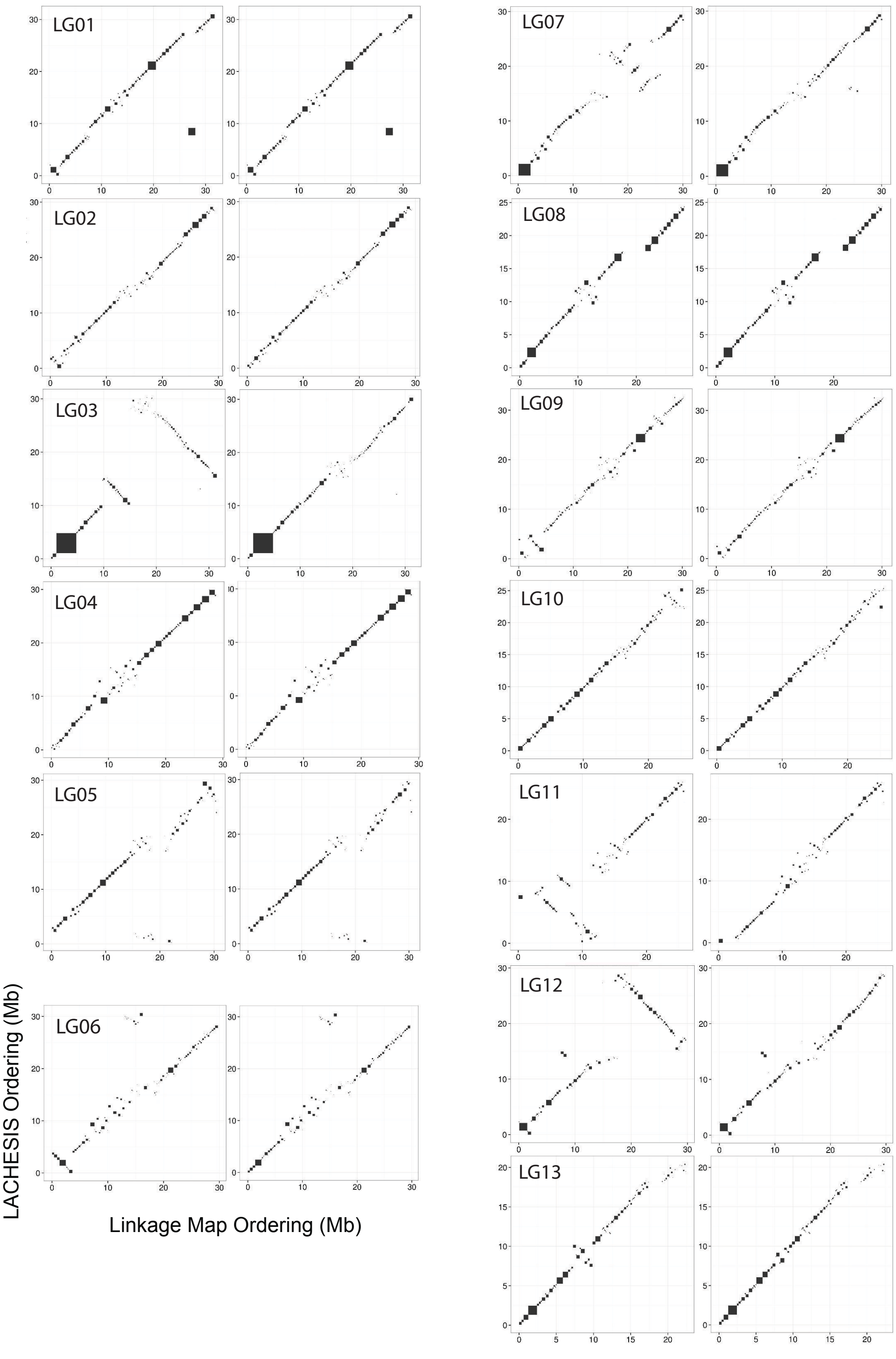
LG01
LG02
LG03
LG04
LG05
LG06
LG07
LG08
LG09
LG10
LG11
LG12
LG13
LACHESIS Ordering (Mb)
Linkage Map Ordering (Mb)

Supplemental Fig. S4. Syntenic dot-plot between *Rhododendron williamsianum* chromosomes. Vertical and horizontal lines delineate chromosomes. Coding sequence from the genome was compared using discontinuous MegaBLAST to identify putative homologous gene pairs. Each dot represents a syntenic gene pair identified by DAGChainer from syntenic blocks with at least 5 gene pairs. Images were generated by SynMap2 within CoGe and can be regenerated at https://genomevolution.org/r/12blb.

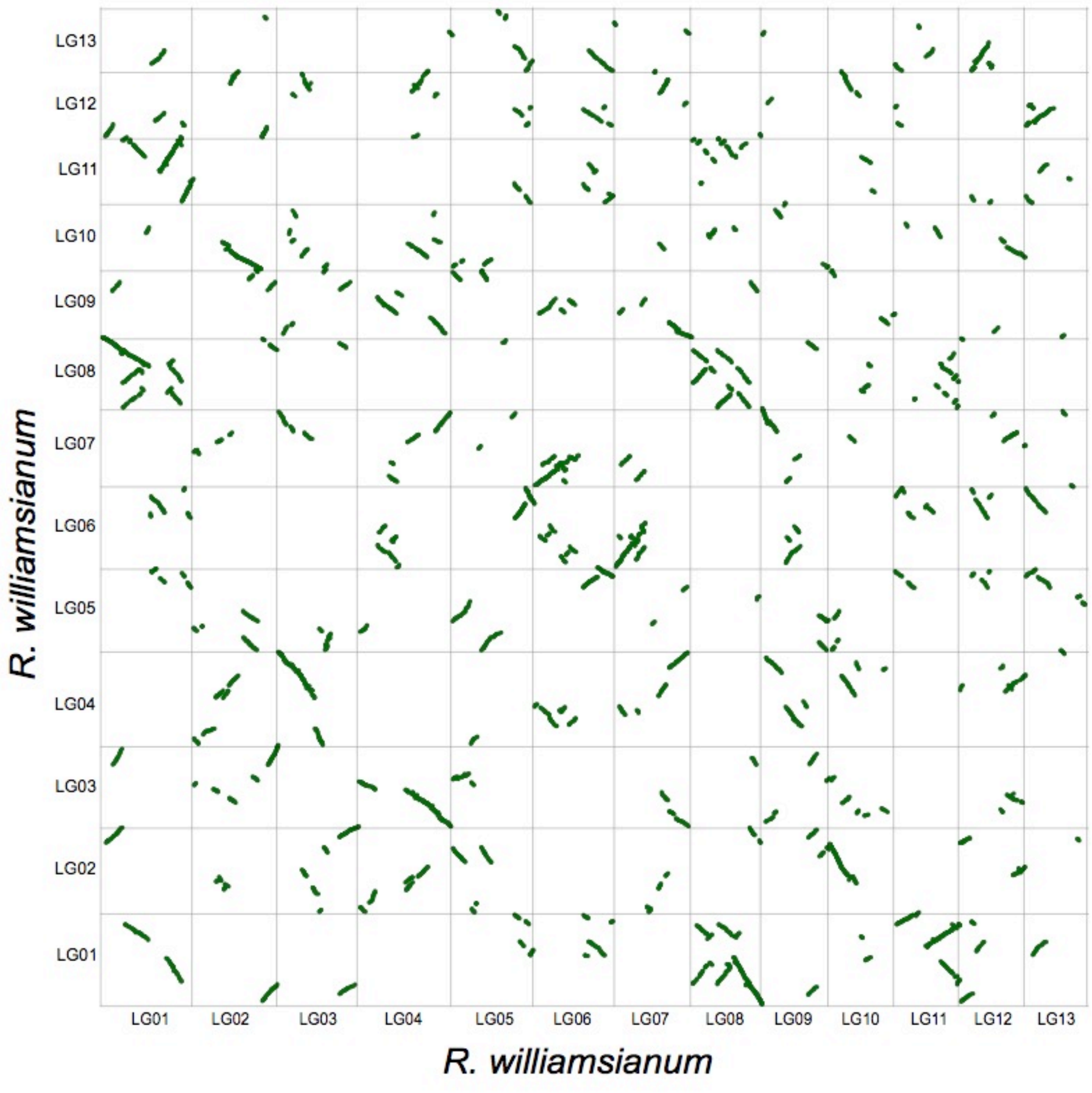

Supplemental Fig. S5. Syntenic dot-plot between *Rhododendron williamsianum* and *Vaccinium macrocarpon* chromosomes. Vertical and horizontal lines delineate chromosomes. Coding sequence from each genome was compared using discontinuous MegaBLAST to identify putative homologous gene pairs. Each dot represents a syntenic gene pair identified by DAGChainer from syntenic blocks with at least 5 gene pairs. Images were generated by SynMap2 within CoGe and can be regenerated at https://genomevolution.org/r/152k5.

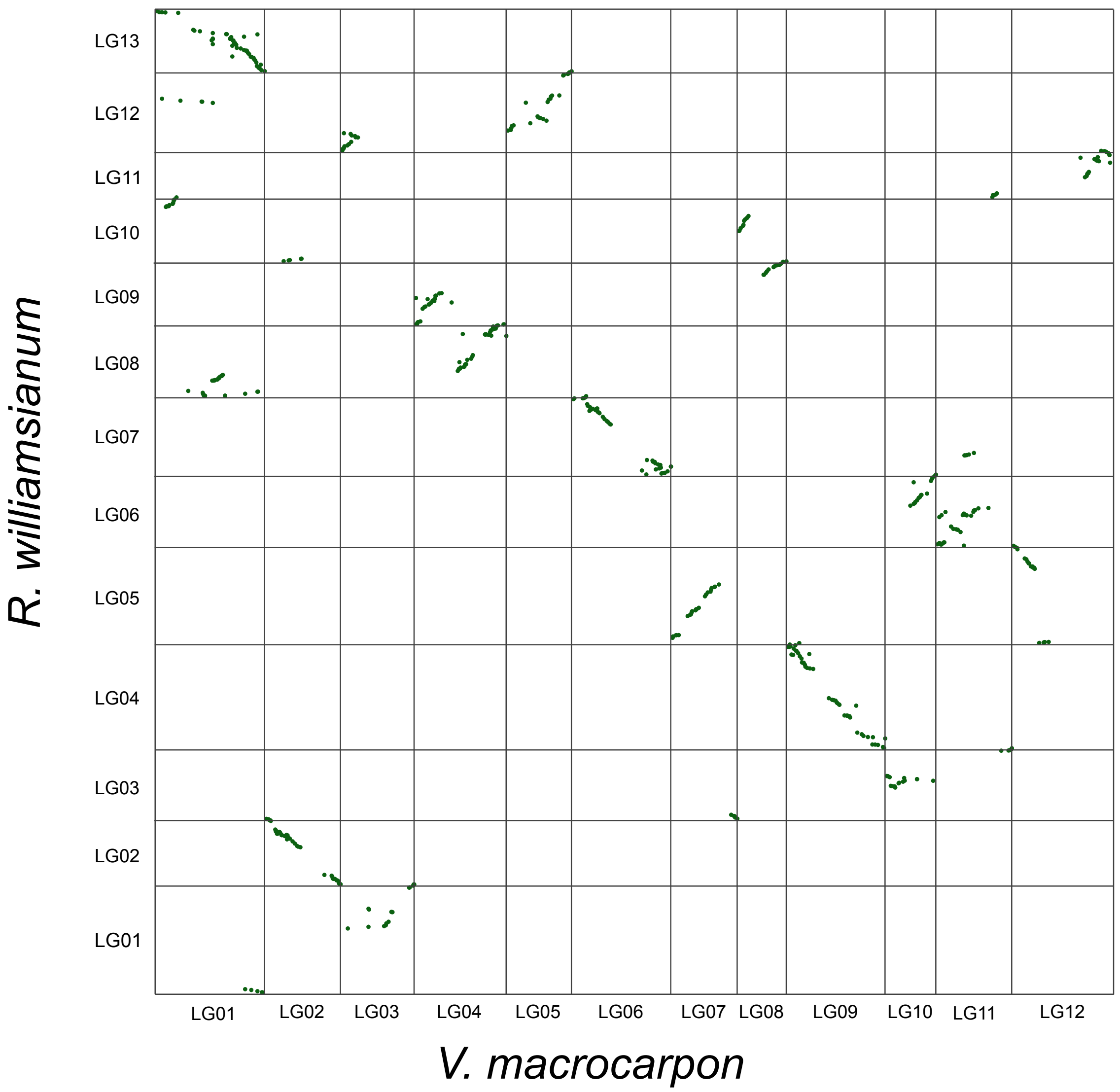

Supplemental Fig. S6. Syntenic dot-plot between *Rhododendron delavayi* scaffolds. Vertical and horizontal lines delineate 193,091 scaffolds. Coding sequence from the genome was compared using discontinuous MegaBLAST to identify putative homologous gene pairs. Each dot represents a syntenic gene pair identified by DAGChainer from syntenic blocks with at least 5 gene pairs. Images were generated by SynMap2 within CoGe and can be regenerated at https://genomevolution.org/r/14kzh.

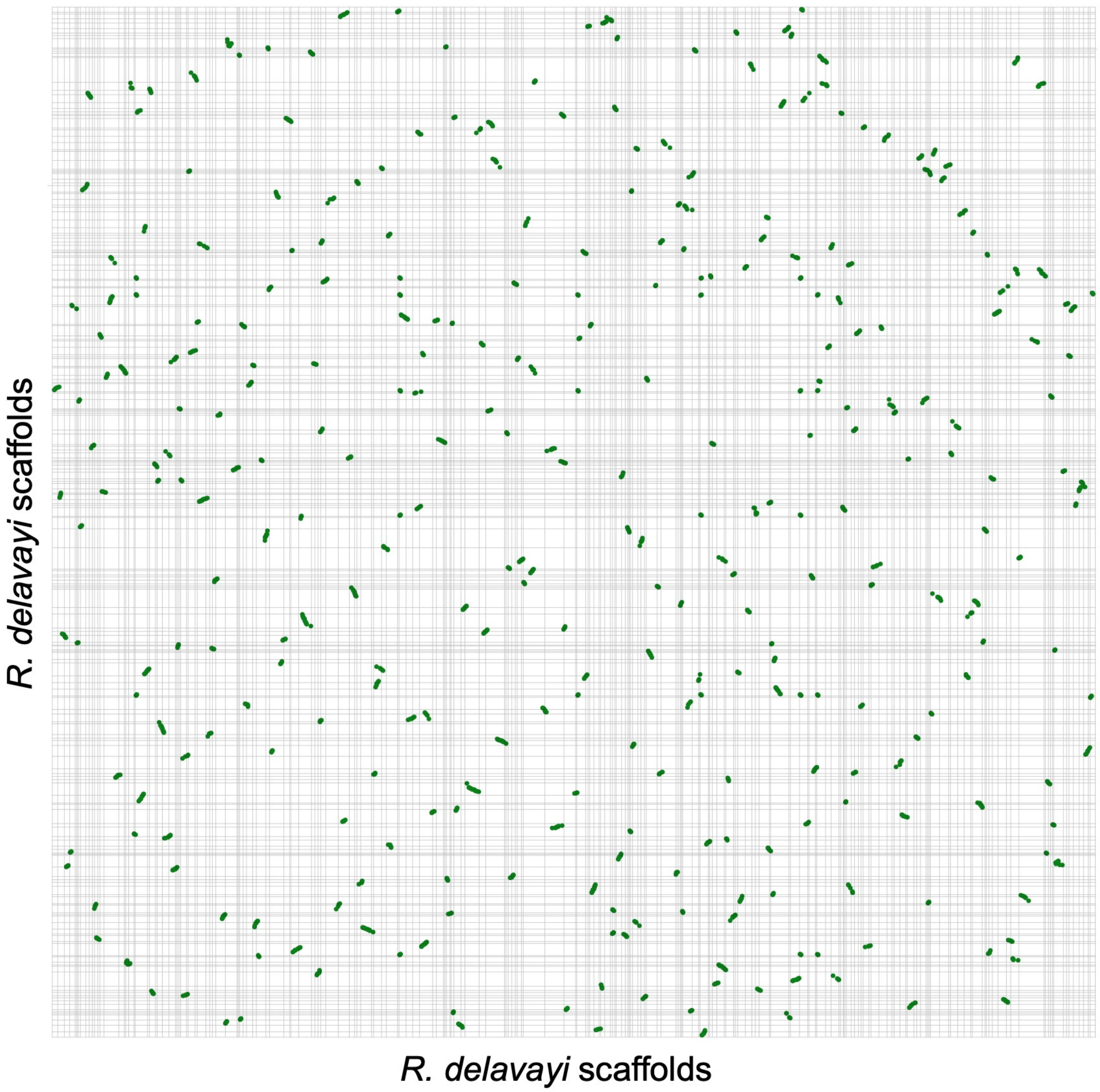

Supplemental Fig. S7. Syntenic dot-plot between *Vaccinium corymbosum* (blueberry) scaffolds. Vertical and horizontal lines delineate 13,757 scaffolds. Coding sequence from the genome was compared using discontinuous MegaBLAST to identify putative homologous gene pairs. Each dot represents a syntenic gene pair identified by DAGChainer from syntenic blocks with at least 5 gene pairs. Images were generated by SynMap2 within CoGe and can be regenerated at https://genomevolution.org/r/14oui.

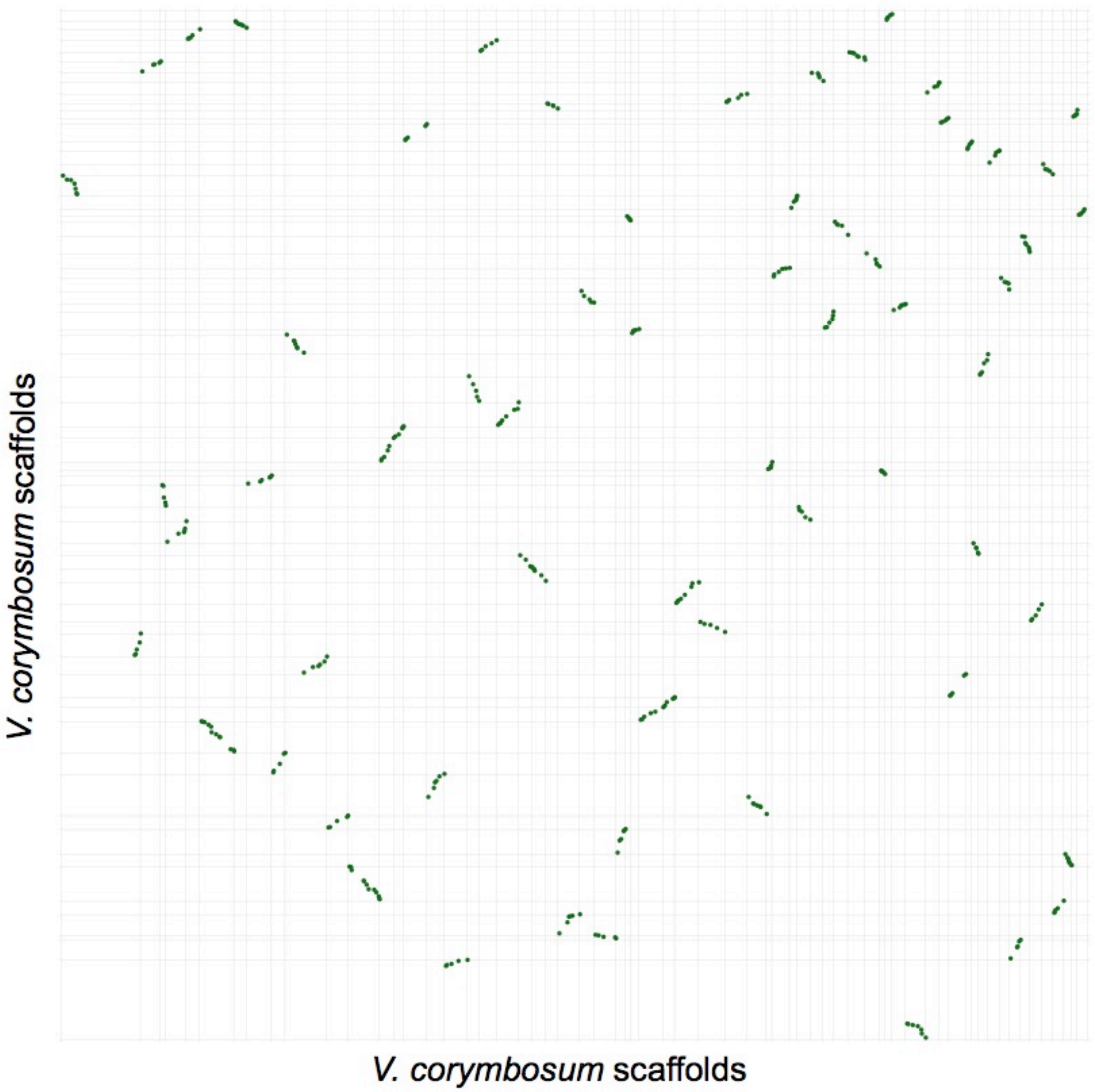

Supplemental Figure S8. Distribution of synonymous substitutions/site (Ks) for paralogous gene pairs in *Actinidia chinensis*. Top panel shows histogram of Ks data overlaid by normal mixture model from EMMIX (McLachlan & Peel 1999). Three components of the normal mixture model are shown in green, red, and blue that correspond with SiZer (Chaudhuri & Marron 1999) results below. Bottom panel shows three significant peaks identified by SiZer map, where blue indicates significant increases and red indicates significant decreases in curves; purple is not significant, gray indicates sparse data.

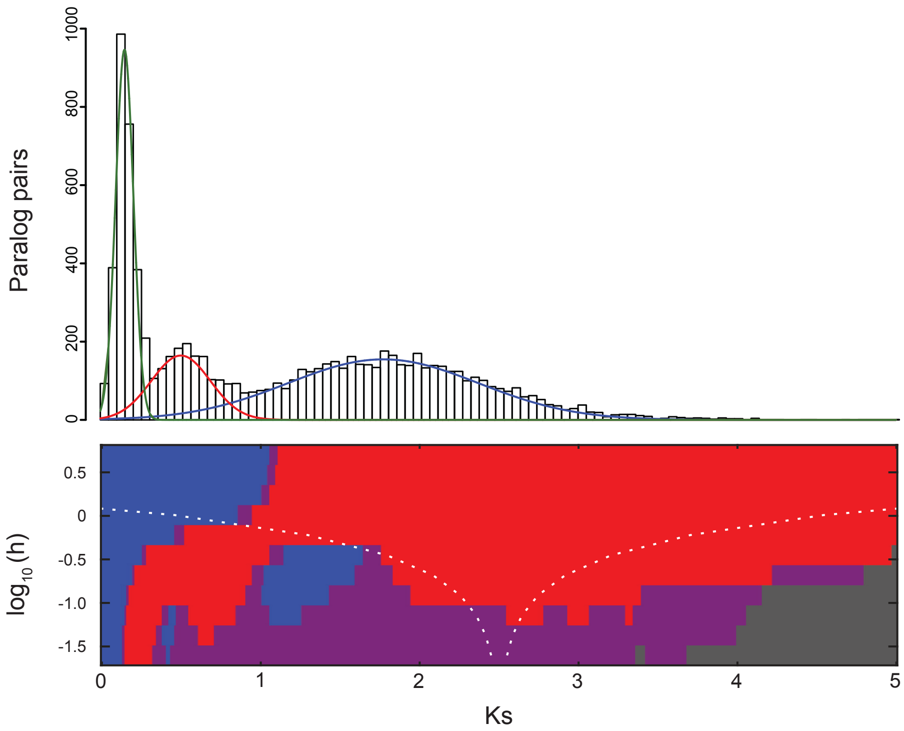

Supplemental Figure S9. Distribution of synonymous substitutions/site (Ks) for syntenic gene pairs in *Rhododendron williamsianum*. Top panel shows histogram of Ks data overlaid by normal mixture model. A normal mixture model with five components was selected by Bayesian Information Criterion in EMMIX (McLachlan & Peel 1999) to estimate parameters of Ks distributions. Red and blue curves correspond to two whole genome duplications found in genomic paralogs of *R. williamsianum*. Bottom panel shows one significant peak identified by SiZer map (Chaudhuri & Marron 1999), where blue indicates significant increases and red indicates significant decreases in curves; purple is not significant, gray indicates sparse data.

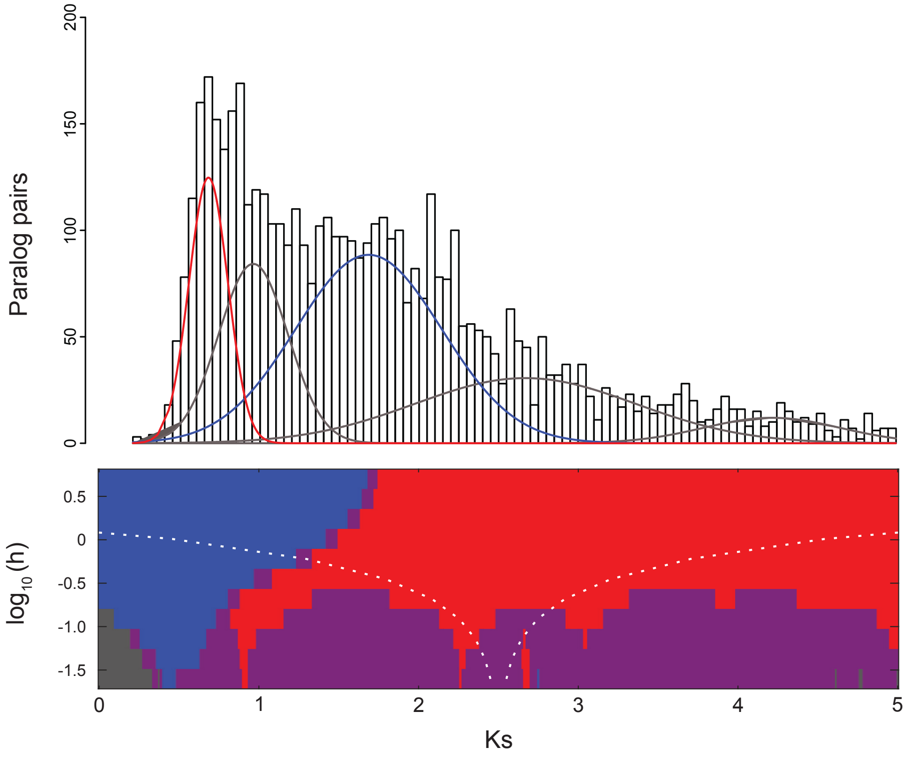

Supplemental Figure S10. Distributions of synonymous substitutions/site (Ks) for orthologous gene pairs between *Rhododendron williamsianum* and relatives. Dark gray histograms are Ks data for paralogous gene pairs in the *R. williamsianum* genome. Red and blue stars indicate whole genome duplications identified in *R. williamsianum*. Light gray histograms are Ks data for orthologous gene pairs between *R. williamsianum* and other genomes: (A) *Vaccinium macrocarpon*, (B) *Actinidia chinensis*, (C) *Camellia sinensis*, (D) *Vitis vinifera*. Dichotomous branches represent speciation events between *R. williamsianum* and other genome represented in each panel.

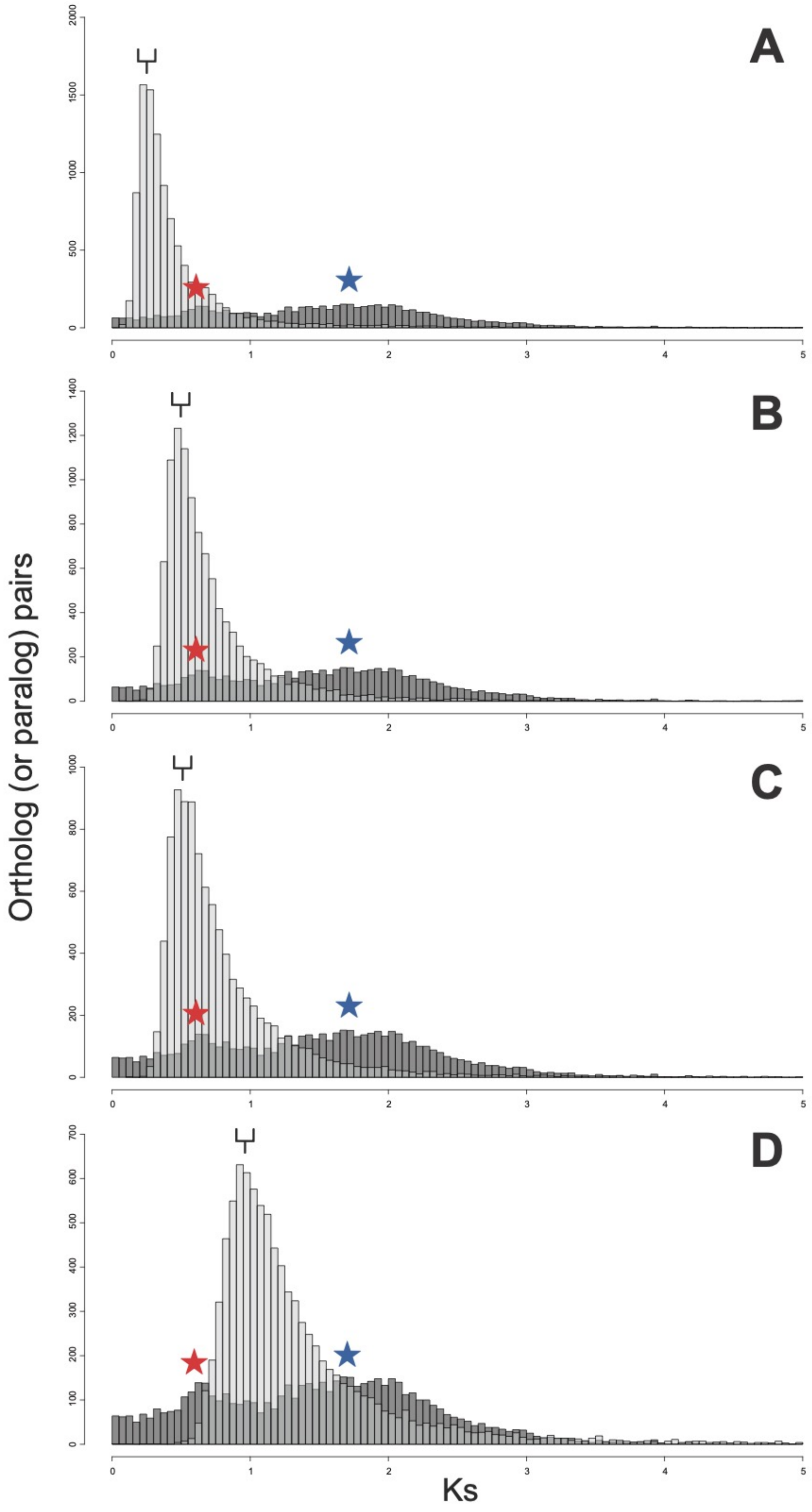
A
B
C
D
Ortholog (or paralog) pairs
Ks
0
1
2
3
4
5
0
500
1000
1500
2000
200
400
600
800
1200
1400
100
300
700
